# IGFBP2 promotes proliferation and cell migration through STAT3 signaling in Sonic hedgehog medulloblastoma

**DOI:** 10.1186/s40478-023-01557-2

**Published:** 2023-04-08

**Authors:** Haritha Kunhiraman, Leon McSwain, Shubin W. Shahab, Timothy R. Gershon, Tobey J. MacDonald, Anna Marie Kenney

**Affiliations:** 1 Department of Pediatrics, Neuro-Oncology Division and Aflac Cancer and Blood Disorders Center of Children’s Healthcare of Atlanta, Emory University, 1760 Haygood Drive, Atlanta, GA 30322 USA; 2 Winship Cancer Institute, Emory University, Atlanta, GA 30322 USA

**Keywords:** Medulloblastoma, Proliferation, Metastasis, Sonic hedgehog, STAT3

## Abstract

Medulloblastoma (MB) is the most common pediatric brain malignancy and is divided into four molecularly distinct subgroups: WNT, Sonic Hedgehog (SHHp53mut and SHHp53wt), Group 3, and Group 4. Previous reports suggest that SHH MB features a unique tumor microenvironment compared with other MB groups. To better understand how SHH MB tumor cells interact with and potentially modify their microenvironment, we performed cytokine array analysis of culture media from freshly isolated MB patient tumor cells, spontaneous SHH MB mouse tumor cells and mouse and human MB cell lines. We found that the SHH MB cells produced elevated levels of IGFBP2 compared to non-SHH MBs. We confirmed these results using ELISA, western blotting, and immunofluorescence staining. IGFBP2 is a pleiotropic member of the IGFBP super-family with secreted and intracellular functions that can modulate tumor cell proliferation, metastasis, and drug resistance, but has been understudied in medulloblastoma. We found that IGFBP2 is required for SHH MB cell proliferation, colony formation, and cell migration, through promoting STAT3 activation and upregulation of epithelial to mesenchymal transition markers; indeed, ectopic STAT3 expression fully compensated for IGFBP2 knockdown in wound healing assays. Taken together, our findings reveal novel roles for IGFBP2 in SHH medulloblastoma growth and metastasis, which is associated with very poor prognosis, and they indicate an IGFBP2-STAT3 axis that could represent a novel therapeutic target in medulloblastoma.

**Supplementary Information:**

The online version contains supplementary material available at 10.1186/s40478-023-01557-2.

## Introduction

Medulloblastoma (MB) is the most common malignant pediatric brain tumor and accounts for approximately 20% of childhood Central Nervous system (CNS) tumors [60]. The current standard of care treatment for MB patients is surgical resection, craniospinal irradiation, and cisplatin-based chemotherapy [49, 62]. These treatments leave patients with life-long neurological and cognitive side-effects, and metastasis is the leading cause of death for medulloblastoma patients, indicating a need for identification of specifically targeted therapies that can ameliorate the need for toxic chemotherapy and radiation treatments and also prevent metastasis.

Based on integrative genomic studies, including both gene expression and methylome profiling, medulloblastoma tumors have been sub-grouped into 4 molecularly distinct categories, including Wingless (WNT), Sonic Hedgehog (SHH), (subdivided into p53 mutant and wild type), Group 3, and Group 4. These subgroups are marked by unique demographics, histological features, chromosomal abnormalities and clinical characteristics, including heterogeneous recurrence patterns and susceptibility for metastatic dissemination [45, 46]. Among the four MB subgroups, the SHH group is the most dominant molecular subgroup in infants and adults [44]. The SHH subgroup comprises almost 30% of all medulloblastomas, with a five-year survival rate of ~ 70% [2, 54, 58]. SHH MB is clinically heterogeneous and can be further divided into four subtypes: SHH-α, SHH-β, SHH-γ and SHH-δ [6]. These tumors are proposed to arise from cerebellar granule neuron precursors (CGNPs), whose developmental expansion is driven by SHH ligand produced by the neighboring Purkinje neurons [8, 66, 69]. Consistent with this origin, a number of SHH pathway targets required for CGNP proliferation are often over-expressed or amplified in SHH MBs, including MYCN, YAP, and Gli1/2 [14, 29, 48, 72].

Recent studies have shown that the tumor microenvironment (TME) plays a major role in regulating tumor growth and metastasis and is a promising therapeutic target. We recently published a report showing that macrophages in the SHH medulloblastoma tumor microenvironment have anti-tumor properties, and that high expression of the macrophage marker AIF1 predicted better survival contrasting with the immune suppressive properties of macrophages in glioma [32, 40]. In addition to the immune component, MB TME includes astrocytes and fibroblasts. Interestingly, recent studies reported that tumor-associated astrocytes (TAA) are crucial components of the MB tumor microenvironment, playing major roles in both primary and relapsed MB progression [19, 38]. We wished to investigate how tumor cells themselves interact with and potentially modulate the TME in medulloblastoma. To this end we asked which signaling molecules are released by MB cells, using cytokine arrays to identify such molecules. Interestingly, we found that freshly isolated SHH patient MB cells, primary mouse SHH MB cells, and SHH MB cell lines produced a number of unique proteins, including elevated levels of insulin-like binding protein 2 (IGFBP2) in comparison to non-SHH patient MB cells and cell lines.

Insulin growth factor 1 (IGF1), Insulin growth factor (IGF2), IGF receptor (IGFR) and IGF Binding Proteins (IGFBPs) are all members of the IGF signaling family and have been implicated in cancer. Expression and activity of IGF1, IGF2 and IGFR are high in primary medulloblastoma tumors and leptomeningeal metastasis [11]. Among the IGFBPs, IGFBP2 expression is higher in the central nervous system (CNS) during the early fetal developmental stage, and it is the second most abundant IGFBP found in circulation [16, 47]. IGFBP2 was originally considered to be a pericellular regulator of IGF1 and IGF2 given that it is secreted from the cell. However, it was later identified as an intracellular and nuclear regulator of tumorigenicity through IGF-independent mechanisms [3, 7]. IGFBP2 is highly expressed in rapidly dividing and motile cells; numerous studies have categorized IGFBP2 as a regulator of cell proliferation, invasion, and metastasis [23, 36, 55, 59]. Finally, increased expression of IGFBP2 is associated with progression in numerous cancers including glioma [35, 42, 57], ovarian [4, 15, 24], prostate [27, 51, 63], pancreatic [1, 28, 41], and breast cancer [68].

Despite the large volume of literature implicating IGFBP2 in growth and metastasis of many types of cancer, there is a dearth of published research on IGFBP2 in medulloblastoma. Indeed, a literature search revealed only two publications: an immunohistochemistry study that reported no IGFBP2 in medulloblastoma, and a gene expression study finding significant-up-regulation of *IGFBP2* in medulloblastoma and a link to poor prognosis [10, 43]. Both studies were small in sample size and did not include molecular subgroup classification, therefore we determined that based on our cytokine array results, the role and function of IGFBP2 in SHH medulloblastoma was worthy of investigation. As described below, our studies in primary mouse SHH medulloblastoma cells and human SHH MB cell lines reveal an essential role for MB cell-derived IGFBP2 in cell proliferation and migration, and we identify STAT3 as a downstream effector of IGFBP2 in regulation of epithelial to mesenchymal transition (EMT) and cell migration, critical steps in metastasis, suggesting a potential for targeting the IGFBP2-STAT3 axis for preventing tumor cell growth and metastasis.

## Materials and methods

### Animal studies

NeuroD2:Smo/A1mice [20, 21] were obtained from Jackson Research Laboratories. The myc-amplified mouse medulloblastoma model was a kind gift of Dr. Robert Wechsler-Reya (Sanford Burnam Prebys) [50]. Breeding, maintenance, and tumor harvest were carried out in compliance with the Emory University Institutional Animal Care and Use Committee guidelines.

#### Microarray analysis

Microarray expression data for medulloblastoma patients was obtained from NCBI Gene Expression Omnibus under accession number GSE85217. Raw data was imported into R Studio followed by analysis using the limma package. R code is available upon request from the corresponding authors**.**

#### Medulloblastoma primary cell culture

Medulloblastoma primary cells (MBCs) were isolated from NeuroD2:Smo/A1 mouse tumors and cultured as described previously [20, 21]. Cells were seeded on Matrigel (Corning) coated plates with Neurobasal medium containing penicillin/streptomycin, 1 mmol/L sodium pyruvate, 1 × B27 supplement, and 2 mmol/L l-glutamine. Primary MBCs were cultured for 24 h before lentiviral infection.

Freshly isolated and frozen patient tumor cells were provided by the Ian’s Friends Foundation Brain Tumor Repository at Children’s Healthcare of Atlanta, Inc. Methylation profiling was used to determine molecular subgrouping. Cells were cultured in Neurobasal: DMEM/F12 GlutaMax Medium (1:1) supplemented with EGF (50 ng/ml), bFGF (40 ng/ml), PDGF- AA (20 ng/ml), PDGF-BB (20 ng/ml), IGF-1 (100 ng/ml), 1X B27 minus vitamin A, heparin (2 µg/ml).

#### Cell lines and cell culture

Human MB cell lines DAOY (p53 mutant) and D283 were originally obtained from ATCC. UW228 (p53 mutant) and ONS76 cell lines were gifted by Dr. Charles Eberhart (Johns Hopkin’s University) and the D425 cell line was a gift from Dr. Eric Raabe (Johns Hopkin’s University). UW228, ONS76 and DAOY are classified as SHH group, D425 is classified as Group 3 and D283 is classified as Group 3/4. ONS76 and UW228 cells were cultured in DMEM/F12 with 10% FBS. DAOY and D283 cells were cultured in EMEM with 10% FBS. D425 cells were cultured in DMEM with 10%FBS. Mouse medulloblastoma-derived PZp53Med cell lines [5] were cultured in DMEM/F12 with 10% FBS. IGFBP2 knockdown stable cell lines were prepared using Control shRNA (pLKO.1 Lentiviral backbone), shGFP, # TRCN0000318716 (sh IGFBP2 #1) (Human IGFBP2 shRNA), # TRCN0000318660 (sh IGFBP2#2) (Human IGFBP2 shRNA), # TRCN0000422736 (Mouse IGFBP2 shRNA), from Millipore Sigma. Knockdown cells were prepared using siRNAs (si STAT3 # 116558) (si IGFBP2 #45934) from Thermo-fisher scientific. Constitutive STAT3 activating cells were prepared using EF.STAT3C.Ubc.GFP plasmid (Addgene plasmid # 24983). IGFBP2 neutralizing antibody (IGFBP2 nAb) and control Goat IgG were applied to the cell lines in serum free medium (2.5–8 µg/ml) (#AF674 R&D systems). Recombinant IGFBP2 (Abcam) was applied to cells cultured in serum free media. STAT3 inhibitor NSC74859 (S3I-201) (# S1155) was purchased from Selleckchem.com.

#### Cytokine array analysis

Human Medulloblastoma cell lines, MB Patient-derived tumor cells, mouse medulloblastoma cell line (PZp53Med cell line) and SmoA1 mouse primary MB cells (MBC) were cultured in serum-free media for 48 h. Conditioned media were collected and analyzed by Human cytokine array kit (C-series Human cytokine array 5 from Ray Biotech) and mouse cytokine array kit (Mouse cytokine array C1000 from RayBiotech) according to the manufacturer’s instructions. Positive controls (a controlled amount of biotinylated antibody printed on the array by manufacturer) were used to normalize the results obtained from different samples. Finally, cytokine profiles were further analyzed by the Microsoft excel sheet provided by RayBiotech.

#### ELISA

The level of secreted IGFBP2 in conditioned media (serum free) was analyzed by Human and mouse IGFBP2 ELISA kit (# ELH-IGFBP2 & #ELM-IGFBP2, Ray Biotech).

#### Western blotting

Tissues and cells were homogenized then lysed in RIPA buffer supplemented with protease inhibitor cocktail and phosphatase inhibitors. 15–30 µg of each sample was denatured and separated in 10% and 12% polyacrylamide gels, then transferred to immobilon-P membranes (Millipore) [30]. Because many of the proteins are of similar molecular weight, parallel gels were run and transferred before cutting membranes for antibody hydridization; equal amounts of protein were loaded in each lane. For quantification purposes, chemiluminescent signals were normalized to that of GAPDH /β-Tubulin on the same blot. The following antibodies were used (IGFBP2 #ab188200 abcam, IGFBP2 #3922S Cell signaling Technology, β-tubulin #SC53140 Santa Cruz Biotechnology, Lamin B1 #13435S Cell signaling Technology, N-Cadherin #13116 Cell signaling Technology, Slug #9585 Cell signaling Technology, E-Cadherin #3195 Cell signaling Technology, MMP-3 #14351 Cell signaling Technology, MMP-7 #71031 Cell signaling Technology, MT1-MMP #13130 Cell signaling Technology, MMP-9 #13667 Cell signaling Technology, STAT3 #12640S Cell signaling Technology, Phospho- STAT3 (Tyr705) #9145 Cell signaling Technology).

#### RNA isolation and quantitative real time PCR

Total RNA from SmoA1 medulloblastomas or cells were isolated using Trizol reagent. 1 µg RNA used for cDNA preparation. cDNA synthesis was performed using High-Capacity cDNA Reverse Transcription kit (Applied Biosystems) according to the manufacturer's protocol. Quantitative Real Time PCR was performed using SYBR Green Master mix (BioRad) in a CFX96 real time PCR machine. List of primers given in the supplementary Table1.

#### Immunofluorescence (IF)

Cell lines were grown on coverslips and MBCs were grown on Matrigel coated coverslips overnight. The cells were fixed with 4% paraformaldehyde for 15 min. Cells were analyzed by immunofluorescence according to standard methods. The primary antibody used for immunofluorescence was IGFBP2 (Abcam), N-Cadherin (Cell signaling Technology). The secondary antibody used was Alexa Fluor 594. Nuclei were stained using DAPI. Cell imaging was performed using a Leica DM2500 microscope equipped with a DFC365FX camera and Leica software. For analysis of paraffin embedded and sectioned NeuroD2:Smo/A1mouse MB samples, slides were deparaffinized, dehydrated and antigen retrieval was performed using Tris–EDTA Buffer, pH 9.0 (Abcam). Tissues were blocked with 5% goat serum. The primary antibodies used for IHC were IGFBP2 (#ab188200 Abcam), GFAP (#3670S Cell signaling Technology). Confocal imaging was performed on the Olympus FV1000 at the Emory University Integrated Cellular Imaging Core.

#### Cell proliferation assay

Cell proliferation was analyzed by CellIiter-Glo assay system (Promega), for which 1 × 10^4^ cells were used.

#### Transwell cell migration assay

Cells (0.1 × 10^6^/well) were seeded on the top of the filter membrane in the Tranwell insert (8 µm pore size) in serum free media. DMEM supplemented with 10% FBS was used as chemo-attractant. After 24 h migrated cells were fixed and stained with 0.2% crystal violet (Sigma Aldrich #V5265). Migrated cells were observed under microscope [31]. Three random fields were imaged, and the number of migrated cells was counted using Image J or cell profiler.

#### Scratch assay

Cells were cultured as monolayers in 24 well plates. After overnight attachment, a 200 µl sterile pipette tip was used to make scratches on the 24 well plate. The floating cells were washed using PBS and further the cells were cultured in FBS free media. The scratch healing was observed under microscope at 0 h, 24 h, 48 h respectively and wound closure was measured using Image J software.

#### 3 D colony formation assay

Cells (5 × 10^2^/well) were seeded in 24 well Matrigel coated plates and incubated with culture media for 15 days. Images were taken in BioTek Lionheart FX and the colony was analyzed by Image J software.

#### Subcellular fractionation

Nuclear and cytoplasmic fractions were separated using subcellular fractionation kits (Thermofisher) according to the manufacturer’s procedure. Cells were harvested using trypsin EDTA solution and washed with ice cold PBS. Proteins were isolated from individual fractions using specific buffers. Individual fractions were analyzed using specific marker proteins like β-Tubulin (Cytoplasmic), Lamin B1 (Soluble nuclear extract) and histone H3 (Chromatin bound nuclear extract).

#### Statistical analysis

Statistical analysis was performed using GraphPad Prism 9. Statistical differences between samples were determined using two sample equal variance student t tests. Measurements were taken from biological replicates. Data were considered as statistically significant if *P* < 0.05.

## Results

### IGFBP2 protein is elevated in the SHH subgroup of medulloblastoma

**Fig. 1:**
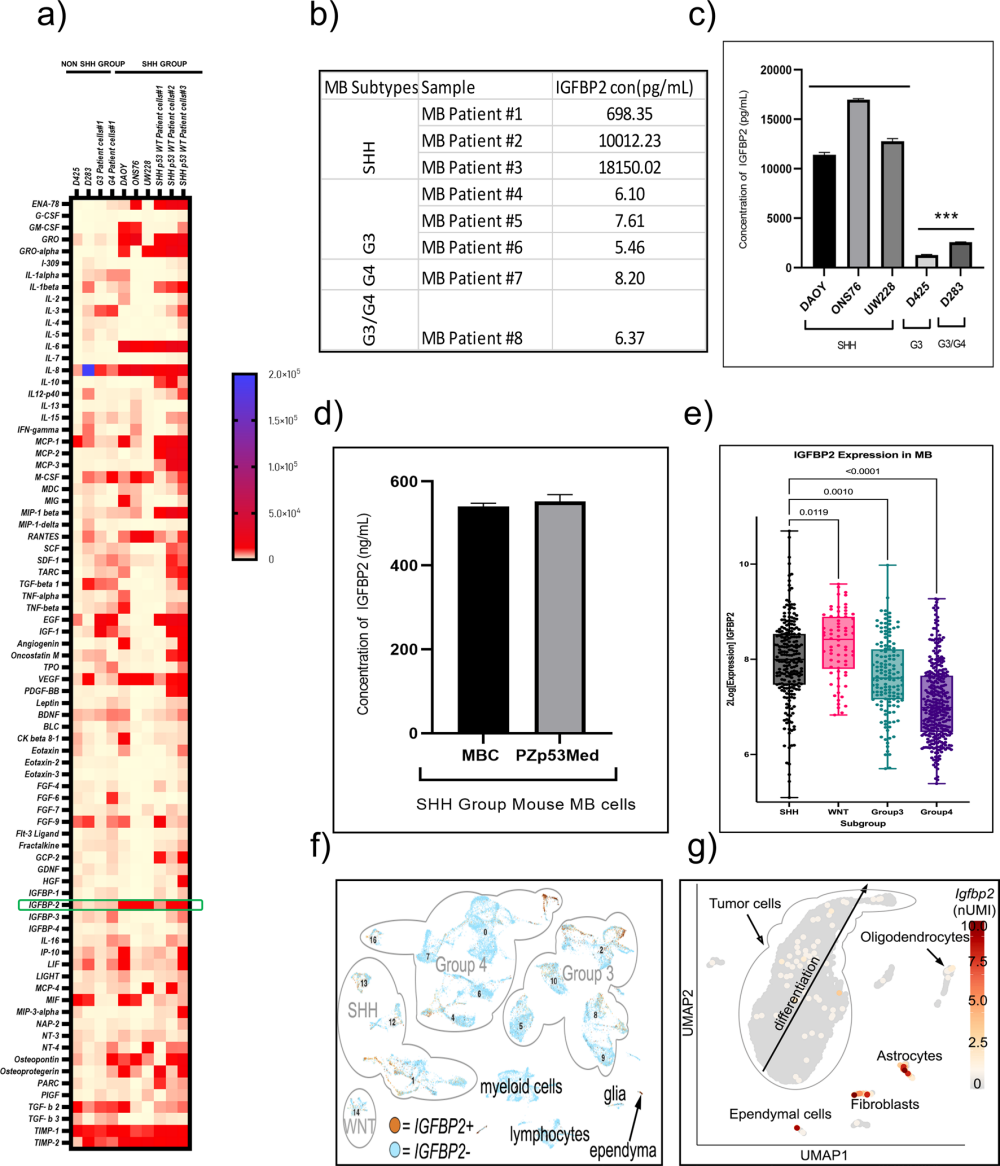
IGFBP2 secretion is increased in SHH Medulloblastoma **a** Heat map showing analysis of secreted proteins in condition media from different human medulloblastoma cell lines (48 h incubation in serum free media) and human MB Patient primary tumor cells. **b** Concentration of IGFBP2 secreted by human mb patient primary tumor cells measured by ELISA. **c** Concentration of IGFBP2 protein secreted by SHH MB human cell lines compared to non SHH MB cell lines analyzed by ELISA. (*n* = 2 independent biological samples) **d** ELISA plot shows IGFBP2 produced by NeuroD2: Smo/A1 mouse primary tumor cells and PZp53 Med mouse mb cell line. (*n* = 2 independent biological samples) **e** Microarray data showing IGFBP2 expression across different MB subgroups (Analysis of Cavalli data set) **f** Analysis of IGFBP2 expression in single cell MB RNA seq data set. **g** UMAP of Math-Cre/smoM2 mutant MB shows IGFBP2 expression in diverse cell types**.** ***P < 0.0003. Data are represented as mean ± S.E.M

To identify differentially secreted proteins in SHH and non-SHH group medulloblastoma, we performed cytokine array analyses of conditioned media from MB cell lines and primary SHH p53 WT MB, Group3 and Group 4 patient cells. While several proteins demonstrated enrichment, IGFBP2 was chosen for further analysis due to higher enrichment levels in SHH MB, its known roles in regulation of tumorigenicity and metastasis in other cancers, and lack of conclusive previous studies in medulloblastoma. Cytokine array analysis of conditioned media from SHH p53 WT MB patient cells showed increased levels of secreted IGFBP2 compared to the non-SHH patient cells (Fig. 1a). Similarly, cytokine array analysis of conditioned media from human SHH group cell lines also showed increased levels of secreted IGFBP2 in SHH group MB compared to D425 and D283, representing groups 3 and 4 MB, (Fig. 1a & Additional file 1: Fig. S1a). We quantified the amount of secreted IGFBP2 in conditioned media by ELISA. The results indicate that the level of secreted IGFBP2 is significantly higher in SHH group MB compared to the non SHH MB (Fig. 1b, c, d).

To determine whether *IGFBP2* is over-expressed in medulloblastoma we re-analyzed microarray data from the [6] medulloblastoma patient dataset and found that the RNA levels of *IGFBP2* are elevated across all MB subgroups (Fig. 1e, *IGFBP2* expression was similar across SHH and non-SHH MB cell lines (Additional file 1: Fig. S1b). The differential levels of protein compared to RNA from our study (Fig. 1e) together with the microarray data support a post-transcriptional regulatory mechanism for IGFBP2 translation. Analysis of RNA single cell sequencing data [39] and qPCR analysis of *IGFBP2* expression in SHH and non-SHH MB cell lines confirmed *IGFBP2* expression in all subgroups, also supporting a post transcriptional mode of protein regulation (Fig. 1f, S1b). Finally, we analyzed the expression of *Igfbp2* in a heterogeneous tumor cell population in mouse SmoM1 mutant MB [39]. Interestingly, UMAP of Smo mutant MB shows *Igfbp2* expression in diverse cell types including astrocytes, fibroblasts, and tumor cells (Fig. 1 g). Taken together, these data suggest a unique role for IGFBP2 protein in SHH MB.

### Subcellular localization of IGFBP2 in SHH MB

**Fig. 2:**
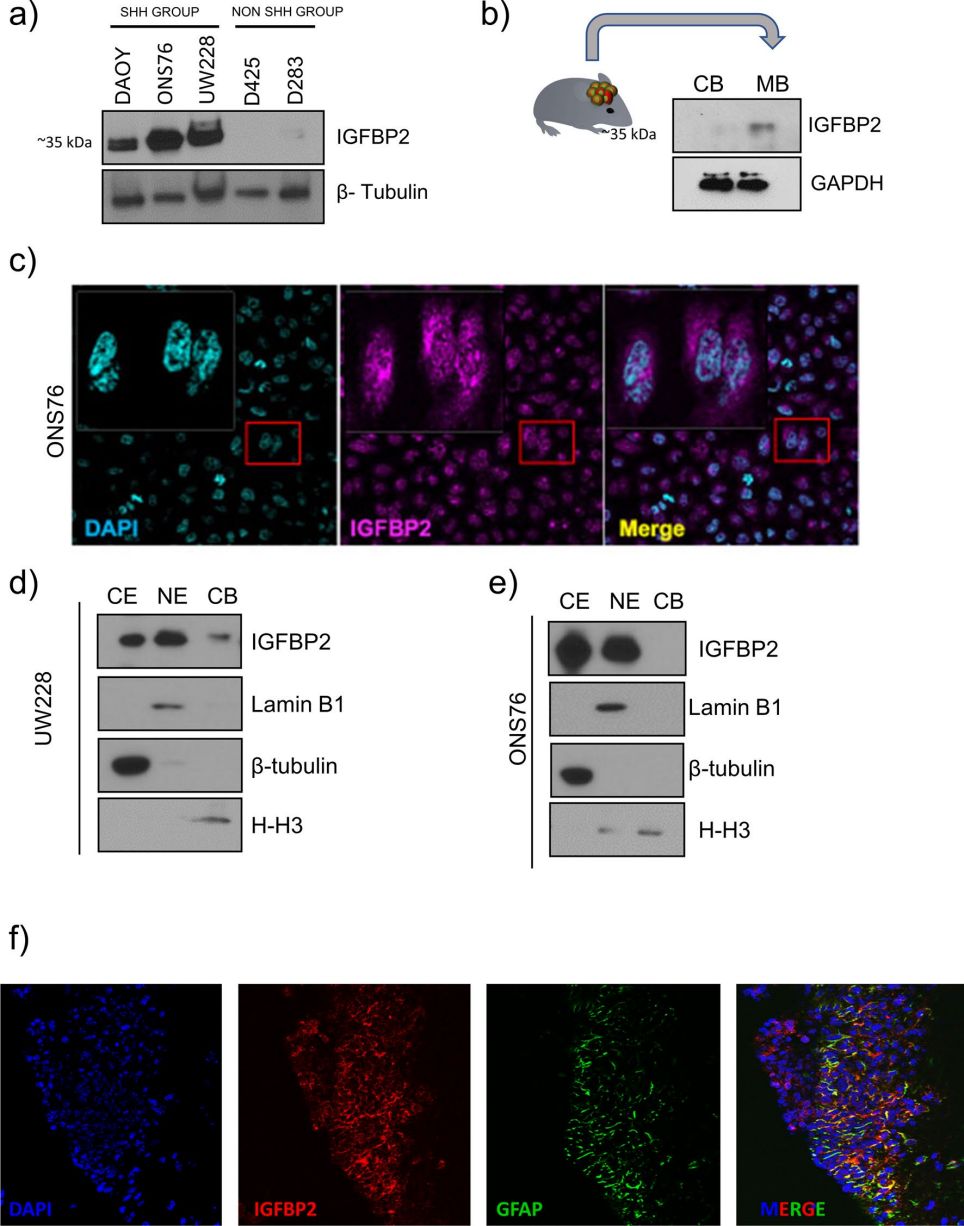
IGFBP2 is present in different cellular compartments **a** Western blot analysis of intracellular IGFBP2 protein in SHH and non-SHH MB cell lines (*n* = 3). **b** Western blot analysis of IGFBP2 in NeuroD2:Smo/A1 MB tumor and non-tumor area (*n* = 4) **c** Analysis of nuclear and cytoplasmic localization of IGFBP2 in ONS76 cell lines by immunofluorescence (*n* = 3). **d**, **e** Western blot analysis of nuclear, cytoplasmic, and chromatin fraction localization of IGFBP2 after subcellular fractionation in SHH MB Cell lines UW228 and ONS76. Controls are tubulin (cytoplasm), Lamin B1 (nucleus) and Histone -H3 (chromatin) (*n* = 3). **f** Immunofluorescence analysis shows IGFBP2 in tumor-associated astrocytes in NeuroD2:Smo/A1 mouse medulloblastoma (*n* = 2)

We used western blot analysis to determine total levels of IGFBP2 in SHH and Non-SHH MB cell lines. As shown in Fig. 2a, levels of IGFBP2 are greater in SHH MB compared to Group 3 and 4. We detected IGFBP2 at ~ 35 kDa molecular weight in Medulloblastoma samples. Next, we confirmed that IGFBP2 upregulation is conserved in mouse models for SHH medulloblastoma, using cytokine array analysis of conditioned media from NeuroD2: Smo/A1 mouse primary medulloblastoma cells and SHH mouse medulloblastoma cell line, PZp53Med (Additional file 1: Fig. S2a, b). We also carried out western blotting of medulloblastoma and adjacent non-tumor tissue from NeuroD2: Smo/A1 mice, and we observed higher levels of IGFBP2 in tumor tissue (Fig. 2b); its levels were very low by comparison in a mouse model for Myc-amplified non-SHH medulloblastoma (Additional file 1: Fig. S2c).

As a secreted protein, IGFBP2 modulates the quantity of IGFs available to bind to the IGF1R, and IGFBP2 can activate signaling in neighboring cells through interactions with extracellular matrix components and cell membrane integrins [33, 34]. However, IGFBP2 also has intracellular functions, including nuclear activity required for its oncogenic functions [3]. We carried out immunofluorescence imaging to determine whether IGFBP2 functions primarily as a secreted factor or has intracellular roles in SHH MB. As shown in Fig. 2c, IGFBP2 is present in both the cytoplasm and nucleus. We confirmed these results using subcellular fractionation, and we also observed a slight accumulation of chromatin bound IGFBP2 in UW228 cell lines (Fig. 2d; these findings were conserved in a mouse SHH MB model cell line (Fig S2d). To support a role for IGFBP2 in the MB tumor microenvironment, and as a confirmation of the single cell sequencing results shown in Fig. 1g, we analyzed the cellular localization of IGFBP2 in mouse MB tissue using immunofluorescence. We observed IGFBP2 throughout the tumor, including in GFAP-positive astrocytes (Fig. 2f). We also observed increased expression of IGFBP2 in SHH Medulloblastoma stem cells (Additional file 1: Fig. S14). These data suggest that IGFBP2 has both secreted and intracellular functions in MB and also plays roles in the SHH MB microenvironment.

### IGFBP2 is required for cell proliferation, colony formation, and cell migration in SHH MB cells

**Fig. 3:**
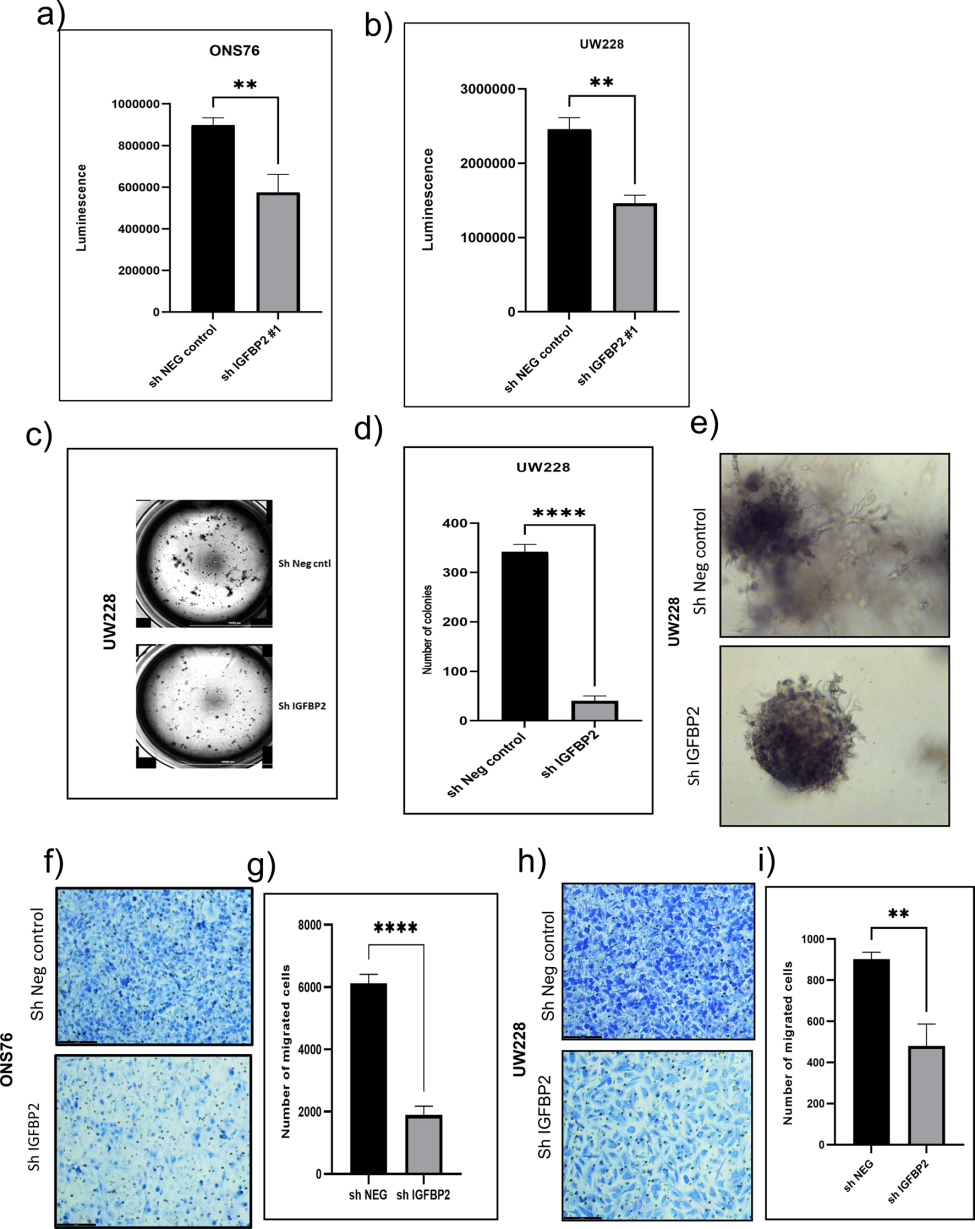
Knockdown of IGFBP2 reduces SHH MBs cell proliferation, colony formation, and cell migration **a** & **b** Cell Titer-Glo analysis of proliferation after IGFBP2 knockdown in ONS76 (**P < 0.0038) and UW228 (**P < 0.0033) MB cell lines (*n* = 3) **c** 3D colony formation assays in wild type or IGFBP2 KD stable cells (*n* = 3) **d** imageJ analysis of colony numbers. ****P < 0.0001. Data are represented as mean ± S.E.M. **e** 3D tumor spheroid formation in control and IGFBP2 knock down cells. **f** & **g** Transwell migration analysis of IGFBP2 knockdown stable cell lines ONS76 and UW228 (*n* = 3). Number of migrated ONS76 (H) and UW228 **I** cells were quantified using image J or cell profiler. (**P < 0.005) Data are represented as mean ± S.E.M

To determine what the function of IGFBP2 is in SHH MB, we established IGFBP2 knockdown stable ONS76 and UW228 cell lines (Additional file 1: Fig.S3a, b, c). First, we studied the effect of IGFBP2 loss on proliferation using CellTiter-Glo assays and 5-Bromo-2-deoxyuridine (BrdU) incorporation assays. We observed that IGFBP2 knockdown significantly reduced cell proliferation compared to the negative control cells (Fig. 3a, b & Additional file 1: Fig. S5d). The number of BrdU positive cells was significantly lower in IGFBP2 knockdown cells compared to the negative control cells (Additional file 1: Fig. S5b, c). In addition, we performed colony formation assays to measure the ability of single cells to grow into larger branching colonies; our results showed that IGFBP2 knockdown cells yielded smaller and fewer colonies compared to the negative controls, with reduced branching (Fig. 3c, d, e). It has been previously reported that IGFBP2 mediates tumor cell metastasis in different cancer types [1, 4, 15, 24, 27, 28, 35, 42, 51, 57, 63]. We also performed *invitro* Transwell migration assays and scratch assays to determine the impact of IGFBP2 knockdown on cell migration. As shown (Fig. 3f, g, h, i & Additional file 1: Fig. S6a, b), loss of IGFBP2 results in significantly reduced numbers of migrated cells compared to the respective negative controls in ONS76 and UW228 cells. And, as shown (Additional file 1: Fig. S4a, b, c & d), loss of IGFBP2 results in significantly reduced wound healing. Taken together, these results indicate that IGFBP2 has important roles in driving SHH MB cell proliferation and migration.

### IGFBP2 is required for expression of epithelial mesenchymal transition (EMT) markers

**Fig. 4:**
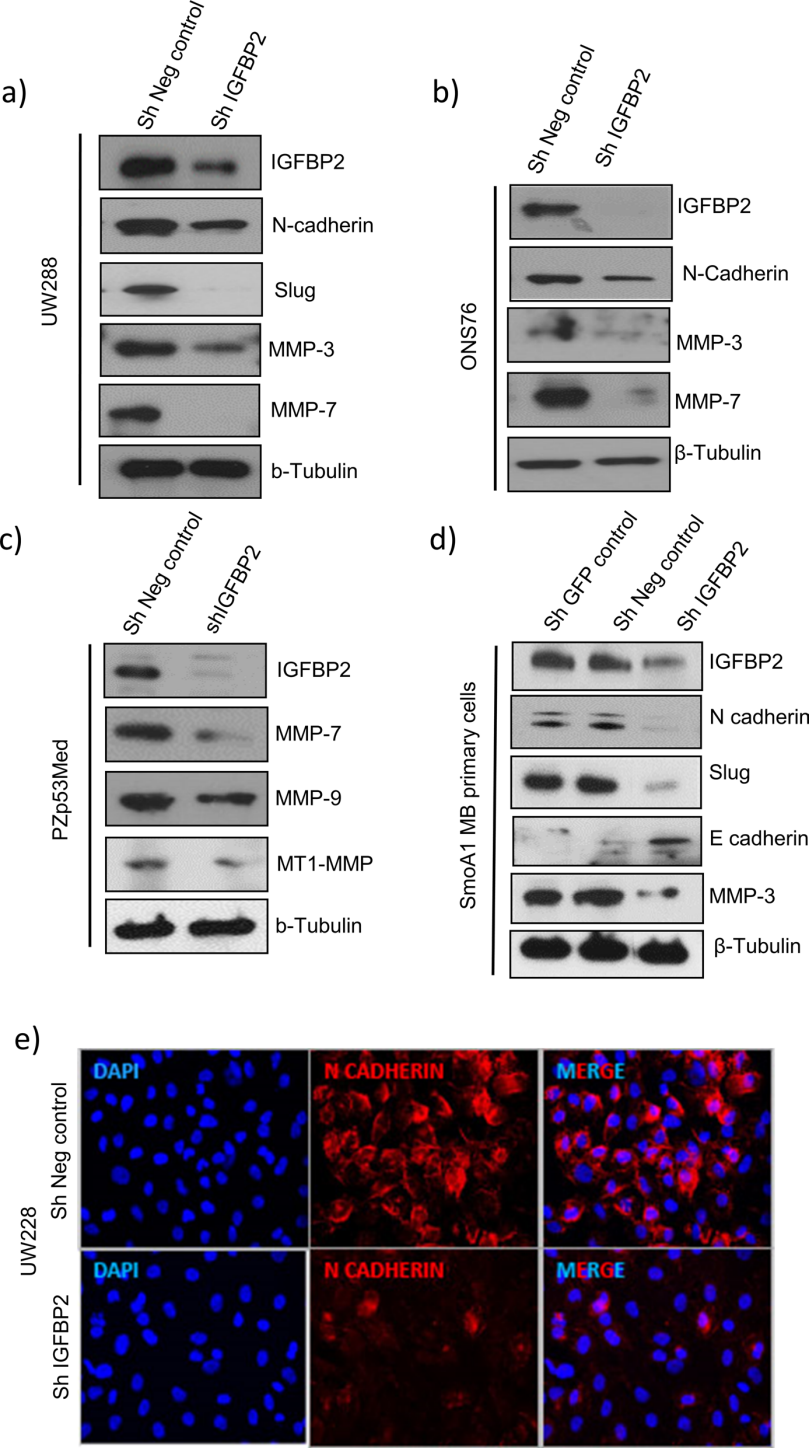
IGFBP2 knock down reduces levels of EMT markers and matrix remodeling marker proteins Representative western blot images of EMT markers and matrix remodeling marker proteins in human and mouse IGFBP2 knockdown stable cell lines **a** UW228 (*n* = 3) **b** ONS76 (*n* = 5) **c** PZp53Med (*n* = 3) and **d** NeuroD2:Smo/A1 mouse primary cells (*n* = 2) **e** Immunofluorescence Analysis of N cadherin in wild type and IGFBP2 knockdown cells (*n* = 3)

Because IGFBP2 knockdown reduced cell migration phenotypes, we used western blotting to analyze the level of expression of select EMT markers in IGFBP2 knock down-cells; these proteins are required for invasion and metastasis in vivo. As shown in Fig. 4, we observed reduced levels of EMT markers such as N-cadherin, slug, and matrix metalloproteinases in both human and mouse SHH mb cell lines and in NeuroD2: Smo/A1 mouse MB primary cells, where we also observed increased levels of E-cadherin, which is essential for cell adhesion and maintaining an epithelial phenotype (Fig. 4a, b, c, d & Additional file 1: Fig. S8a, b, c, d). Immunofluorescence analysis of UW228 IGFBP2 knockdown and control cells confirmed down-regulation of N-cadherin (Fig. 4e, Additional file 1: Fig. S7). Knockdown of IGFBP2 also reduces expression levels of mRNA of mesenchymal marker genes such as N-Cadherin and snail and also increased the expression of epithelial marker genes such as E cadherin (Additional file 1: Fig. S3d). Taken together, these results support a role of IGFBP2 regulation of EMT in SHH MB.

### STAT3 lies downstream of IGFBP2 in regulating EMT markers and cell migration

**Fig. 5:**
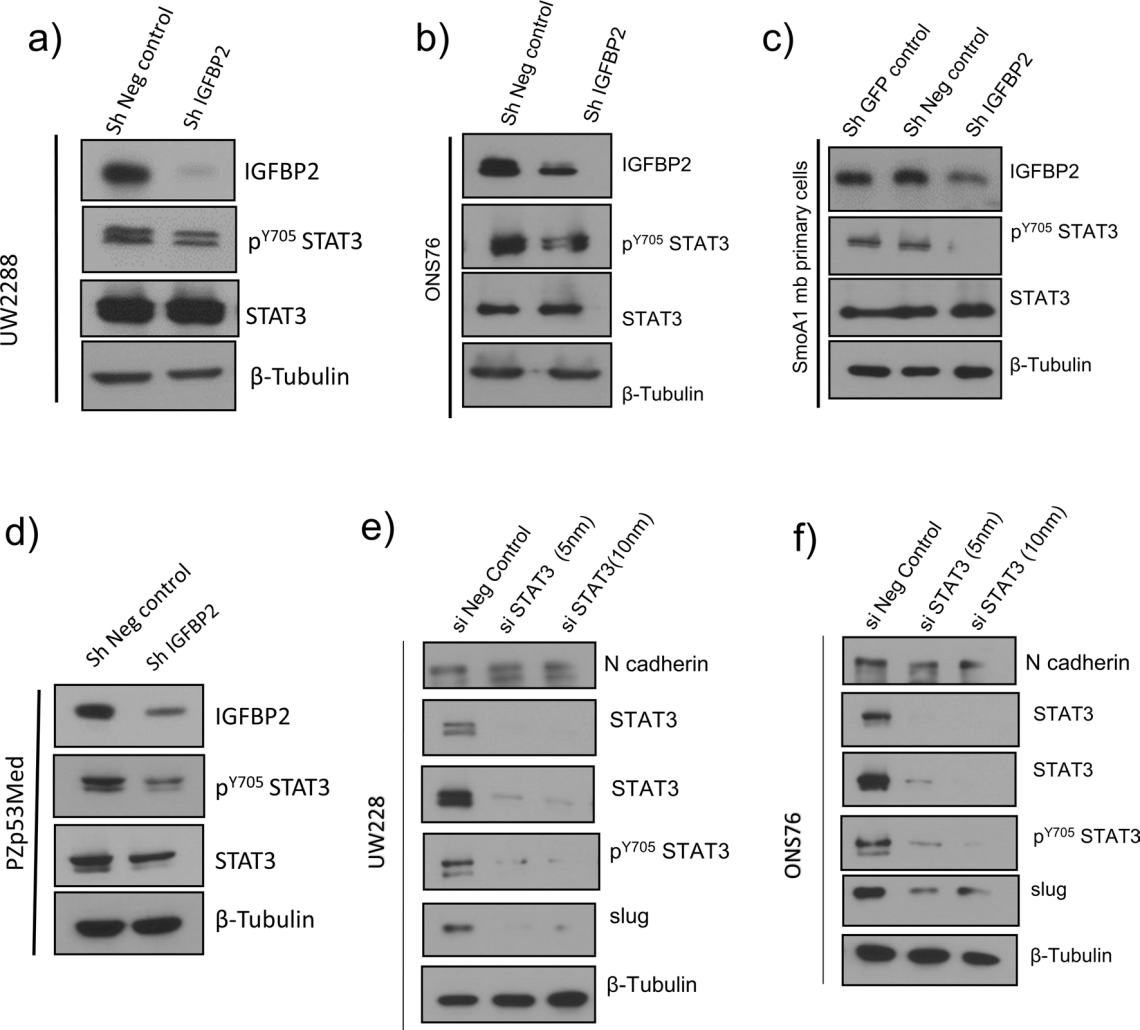
IGFBP2 knockdown reduces STAT3 activating phosphorylation Representative western blot images STAT3 phosphorylation in IGFBP2 knockdown stable cell lines **a** UW228 (*n* = 3) **b** ONS76 (*n* = 5) **c** PZp53Med (*n* = 3) and **d** NeuroD2: Smo/A1 mouse primary MB cells (*n* = 2). Western blot analysis of STAT3 phosphorylation and EMT markers after treatment with si STAT3 in **e** UW228 & **f** ONS76 cell lines (*n* = 3)

Having established that IGFBP2 reduces SHH MB tumor cell migration and levels of EMT markers, we next wished to determine the signaling mechanisms downstream of IGFBP2 regulating these phenotypes. Several studies have reported a link between IGFBP2 and STAT3 activity and STAT3 is known to play essential roles in regulating EMT [22, 33, 34]. We therefore analyzed the activation of STAT3 in IGFBP2 knockdown stable cell lines and primary mouse MB cells by western blot analysis. These results indicated that there is no change in total STAT3 protein in the presence and absence of IGFBP2. However, IGFBP2 knockdown significantly reduces STAT3 phosphorylation at tyrosine 705 (Y705) compared to the respective negative controls (Fig. 5a, b, c, d; Additional file 1: Fig. S3a and S9a, b, c, d); Y705 phosphorylation of STAT3 is key for its transcriptional activation [25]. To confirm the role of STAT3 in the EMT process in medulloblastoma cells, we performed STAT3 knockdown using STAT3 siRNA and then analyzed the expression of EMT markers in those cells. Our results revealed that STAT3 knockdown decreased levels of mesenchymal markers such as N-cadherin and slug compared to the control cells (Fig. 5 e, f & Additional file 1: Fig. S10a, b).

**Fig. 6:**
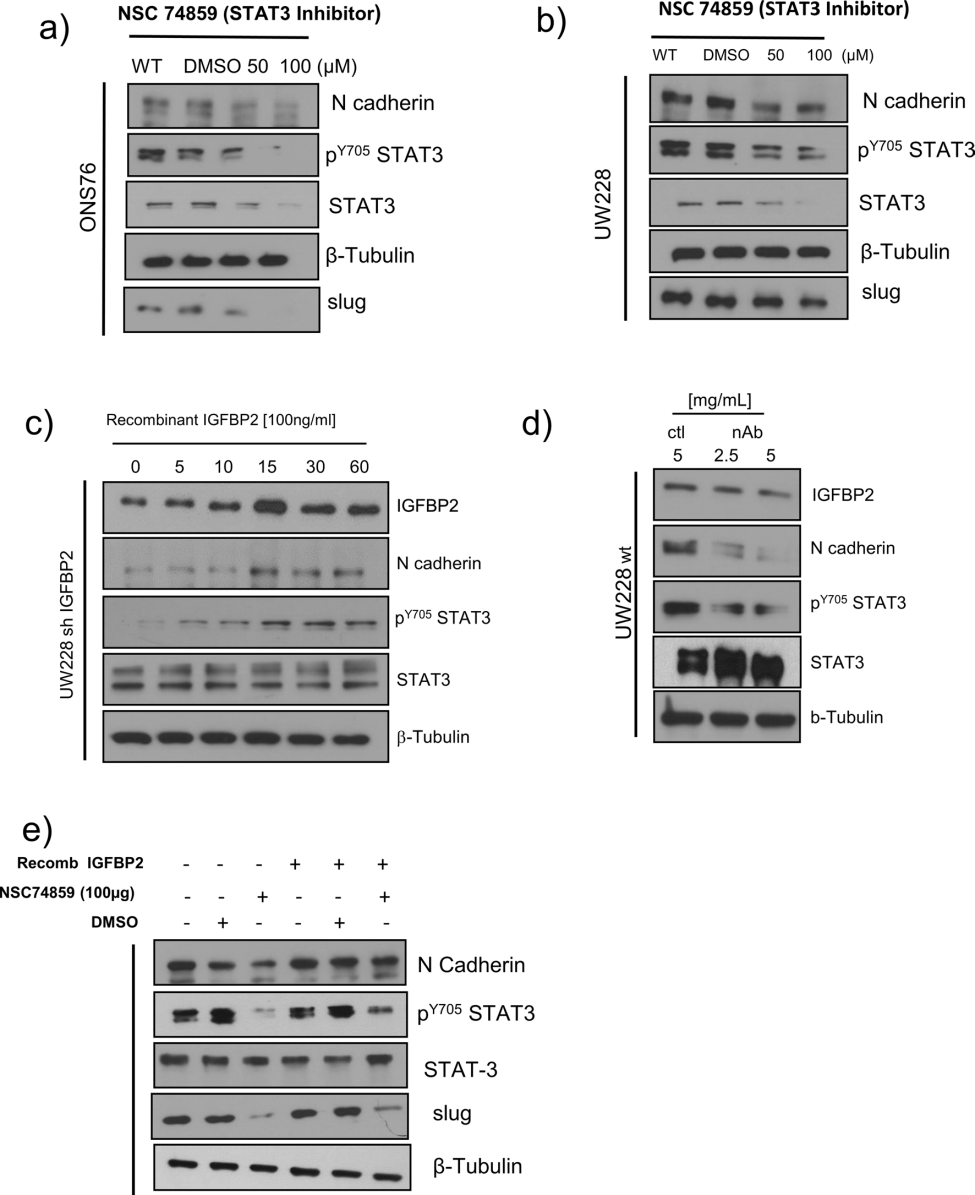
Exogenous IGFBP2 modulates STAT3 activity Western blot analysis of STAT3 phosphorylation and EMT markers **a** ONS76 **b** UW228 after treatment with NSC74859 for 48 h (*n* = 3) **c** Western blot analysis of STAT3 phosphorylation and EMT markers levels in IGFBP2 knockdown stable cells (UW228 shIGFBP2) cultured in serum free condition overnight then treated with exogenous IGFBP2 (100 ng/ml) for the specified time points (*n* = 2) **d** Western blot analysis of STAT3 activation and N-cadherin levels in UW228 cells treated with control IgG or IGFBP2 neutralizing antibodies (*n* = 2) **e** Western blot analysis of STAT3 phosphorylation and EMT marker levels in ONS76 cells treated with NSC74859 for 48 h and then treated with exogenous IGFBP2 (100 ng/ml) for 1 h (*n* = 3)

Next, we tested the effect of a STAT3 inhibitor (NSC74859) on EMT markers in SHH MB cells. To that end, we treated SHH MB cells with NSC74859 at different concentrations for 48 h. We found that NSC74859 inhibited p-STAT3(Y705) in SHH MB cells in a dose dependent manner and reduced the levels of EMT markers N-cadherin and slug compared to the DMSO control (Fig. 6a, b & Additional file 1: Fig. S11a, b). We also analyzed STAT3 and STAT3 Y705 phosphorylation in IGFBP2 knockdown cells after stimulation with exogenous IGFBP2. We found that IGFBP2 stimulation of these cells caused no change in STAT3 levels, but the level of STAT3 Y705 increased over time, along with N-cadherin (Fig. 6c & Additional file 1: Fig. S12a). Conversely, treatment of wild type cells with IGFBP2 neutralizing antibodies resulted in reduced levels of N-cadherin and STAT3 Y705 phosphorylation (Fig. 6d & Additional file 1: Fig. S12b). We next wished to confirm that IGFBP2 plays a role in STAT3-mediated EMT marker regulation. We treated SHH MB cells with NSC74859 for 48 h, then removed the media and added recombinant IGFBP2 (100 ng/ml) for 1 h followed by analysis of STAT3 Y705 phosphorylation and EMT markers. We found that IGFBP2 reverses STAT3 phosphorylation inhibition and rescues EMT markers (Fig. 6e & Fig. S13a). Taken together, these results suggest that exogenous IGFBP2 signals to promote STAT3 Y705 phosphorylation and downstream EMT protein upregulation.

**Fig. 7:**
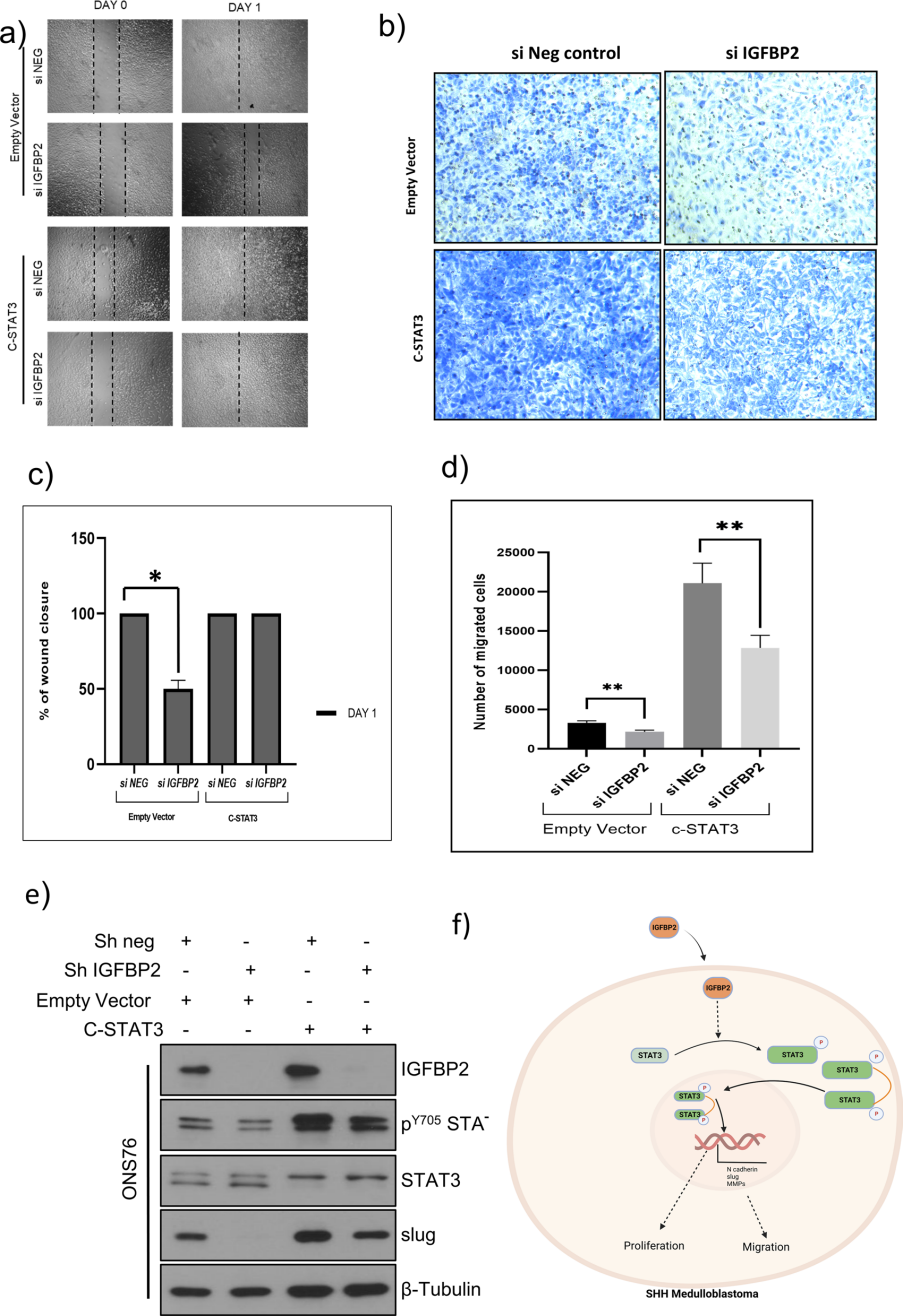
Constitutively active STAT3 (C-STAT-3) rescues wound healing, cell migration and EMT markers in IGFBP2 knockdown cells **a** Scratch assay analysis of ONS76 cells expressing vector control or constitutively activated STAT3 and in the presence or absence of IGFBP2 (*n* = 3). **c** Percentage of wound closure by ONS76 cells measured by image J. **b** Transwell migration assay of ONS76 cells expressing vector control or constitutively activated STAT3 and in the presence or absence of IGFBP2 (*n* = 3) **d** Quantification of cells migrated measured by image J. **e** Western blot analysis of EMT markers in cells expressing vector control or constitutively activated STAT3 and in the presence and absence of IGFBP2. Data are represented as mean ± S.E.M. (**P < 0.005) **f** Schematic illustrating IGFBP2-STAT3 signaling axis regulating proliferation and migration in medulloblastoma cells

To determine whether STAT3 functions downstream of IGFBP2 in promoting wound healing, we performed rescue experiments wherein we expressed a constitutively active STAT3 (C-STAT3) in IGFBP2 knockdown SHH MB cell lines, then carried out scratch assays, migration assays and western blotting for downstream markers of EMT. As shown in Fig. 7a,b, c, d, e & Additional file 1: Fig. S13b, C-STAT3 was able to rescue wound closure in scratch assays, cell migration and expression of the EMT marker slug. These data suggest that IGFBP2 mediates cell migration and EMT maker upregulation through STAT3 phosphorylation and activation in vitro and suggest that an IGFBP2 → STAT3 signaling axis that could drive invasion and metastasis in vivo.

## Discussion

Here we report elevated levels of IGFBP2 protein in the SHH group of medulloblastoma based on our studies in MB patient tumor cells, human and mouse MB cell lines and mouse primary MB cells. It has been previously reported that *IGFBP2* and *IGFBP3* mRNA expression is elevated in medulloblastoma and correlates with poor prognosis [10]. However, another report from Nararayan R et al. suggested that IGFBP2 is absent in MB based on immunohistochemistry of patient samples. We speculate that this study, which utilized adult glioblastoma as a positive control, may not have detected the expression of alternate IGFBP2 isoforms in pediatric MB. Moreover, the above studies were of limited sample size and did not include molecular subgroup classification, potentially even excluding SHH MB patients.

Our comprehensive analysis of large medulloblastoma patient gene expression and single cell RNA sequencing datasets indicate that *IGFBP2* is expressed across all molecular subgroups of medulloblastoma, indicating a post-transcriptional mode of regulation driving elevated IGFBP2 protein in SHH medulloblastoma. Although microarray data show that WNT MB has the highest expression of IGFBP2, WNT MBs have the most favorable prognosis and this is the only MB subgroup in which metastasis is not an indicative of poor prognosis [53, 65]. IGFBP2 is a member of a family of six IGF-binding proteins, whose primary function is thought to be modulating the activity of the IGF pathway by binding to IGF1 and 2 and regulating their access to the IGF1 receptor. They also function to prevent IGF binding to the insulin receptor, which would lead to hypoglycemic effects due to the high concentration of circulating IGF. In addition to modulating IGF signaling, IGFBPs have independent functions, including interacting with integrins to regulate cell migration and adhesion, binding to receptor tyrosine phosphatases, and nuclear transactivation properties [33, 34]. The IGFBPs have much common sequence homology but each also possesses unique properties, for example IGFBP2 has a heparin binding domain (HBD) that overlaps with its nuclear localization sequence, which interacts with Importin-α [70].

IGFBP2 has been shown to play oncogenic and pro-migration roles in numerous types of cancer, including glioma, breast, liver, leukemia, gastric, and bladder. Elevated levels of expression predict poor prognosis and are associated with greater malignancy in adult and pediatric cancers [7, 33, 34, 64]. In glioblastoma, nuclear IGFBP2 leads to aberrant EGFR and STAT3 signaling in vitro, and RCAS-mediated *IGFBP2* expression increased glioblastoma formation and progression in vivo in a mouse model, which was reversed when *IGFBP2* expression was inactivated [13]. Neutralizing antibodies against IGFBP2 blocked glioma cell growth in vitro and in vivo, demonstrating the importance of IGFBPs in these tumors [37, 52]. IGFBP2 interactions with integrin- α5 promote migration in glioma and Ewing’s sarcoma [56, 67]. IGFBP2 also promotes STAT3 activity in prostate cancer invasion and promotes NF-κB-driven invasion in pancreatic cancer [9, 17]. IGFBP2 itself has proven difficult to target with drugs, hence the focus on identifying druggable downstream effectors such as STAT3, NF-kB, and integrin-linked kinase.

Consistent with its pleiotropic functions, we found that in addition to being secreted, IGFBP2 is present in cytoplasmic, nuclear, and chromatin subcellular fractions of SHH MB. Single cell RNA sequencing analysis of Smo mutant mouse medulloblastoma shows IGFBP2 expression in tumor cells and microenvironment components including astrocytes, oligodendrocytes, and perivascular fibroblasts. Future studies using cell- and tissue-specific IGFBP2 ablation will lead to insight as to how IGFBP2 produced by each of these cell types contributes to the overall tumor milieu; the present study focuses on the function of IGFBP2 produced by medulloblastoma tumor cells.

In our study, we found that IGFBP2 knockdown cells feature reduced proliferation and smaller colony size and number compared to negative control cells. Moreover, reduced wound closure and Matrigel colony spread after IGFBP2 knockdown also suggest a potential role for IGFBP2 in SHH MB metastasis. Overall, mechanisms driving MB metastasis are poorly understood. Until recently, it was thought that medulloblastoma spread to the leptomeninges took place by a passive diffusion through the cerebral spinal fluid. However, the recent identification of circulating tumor cells and the elegant report from the Taylor group showing that in a mouse model driven by activated SHH pathway signaling, tumor cells may take a hematogenous route to leptomeningeal and non-CNS spread indicates that metastasis is a very active process [18].

Epithelial Mesenchymal Transition (EMT) is a reversible cellular program in which polarized epithelial cells progressively obtain mesenchymal character [12, 26]. Because of EMT, cells lose polarity and cell–cell adhesion, acquiring migratory and invasive properties. EMT is an important process during embryogenesis, wound healing and also in tumor progression [12]. IGFBP2 is known to promote EMT in a variety of tumors, including pancreatic ductal adenocarcinoma, glioma, and prostate cancer [17, 36–38]. In addition to phenotypic changes, our results also reveal changes in levels of various EMT markers and extracellular matrix remodeling proteins, required for loss of adhesion and subsequent cell migration.

Several studies have shown a link between IGFBP2 and STAT3 activity, and STAT3 is known to regulate cell proliferation and EMT phenotypes [7, 33, 34, 61, 71]. STAT3 is phosphorylated at tyrosine705 by Janus Kinase family members, which stimulates its homodimerization and nuclear localization, and transcription of EMT-promoting genes. When we analyzed STAT3 phosphorylation in wild type and IGFBP2 knockdown MB cells, we observed that STAT3 is constitutively active in control MB cells and loss of IGFBP2 resulted in down-regulation of STAT3 phosphorylation at Y705. Our findings suggest that in SHH medulloblastoma, as in glioma [7], IGFBP2 may regulate a STAT3-mediated EMT program in order to drive metastasis. In glioma, IGFBP2 forms a complex with the EGFR leading to EGFR accumulation inside the nucleus and induction of STAT3 transactivation [7]. Whether such a mechanism is conserved in medulloblastoma remains to be determined; our immunostaining and fractionation assays indicate substantial IGFBP2 accumulation in the nucleus of mouse and human medulloblastoma cell lines, but extracellular IGFBP2 signaling promotes and is required for the effects of IGFBP2 on STAT3 phosphorylation and EMT marker expression, as exposure of IGFBP2 knock-out mb cells to recombinant IGFBP2 results in rescue of STAT3 phosphorylation and N-cadherin protein, while treatment of wild type MB cells with IGFBP2 neutralizing antibodies has the opposite effect.

In summary, our results using patient samples, primary MB cells isolated from genetically engineered mouse models, and mouse and human MB cell lines reveal IGFBP2 protein upregulation in SHH group MB and provide evidence that IGFBP2 plays an essential role in tumor cell proliferation, migration, STAT3 activity, and EMT marker levels in SHH MB, roles not conserved in non-SHH mb which have low levels of IGFBP2 protein (Additional file 1: Fig. S15). Future studies will focus on elucidating the therapeutic potential for blocking IGFBP2 and/or STAT3 in vivo to reduce SHH medulloblastoma tumor growth and prevent metastasis, thereby improving patient survival and quality of life, as well as perhaps permitting reduced exposure to radiation and toxic chemotherapies given the established roles of IGFBP2 in resistance to therapy in other types of cancer.

## Supplementary Information

**Additional file 1.** Supplementary data file.

## Data Availability

The human single cell sequencing dataset analyzed in the studies is publicly available at http://pneuroonccellatlas.org. The mouse single cell sequencing data set can be accessed at https://github.com/malawsky/Gershon_single-cell. Microarray expression data for medulloblastoma patients was obtained from NCBI Gene Expression Omnibus under accession number GSE85217.
