# Supplementary material for: IGFBP2 promotes proliferation and cell migration through STAT3 signaling in Sonic hedgehog medulloblastoma
Source: Acta Neuropathol Commun. 2023 Apr 8;11:62. doi: 10.1186/s40478-023-01557-2 (PMC10082504; doi:10.1186/s40478-023-01557-2)
Supplement: Supplementary file 1 — Additional file 1. Supplementary data file. [file 40478_2023_1557_MOESM1_ESM.pdf]

Kunhiraman Fig S1

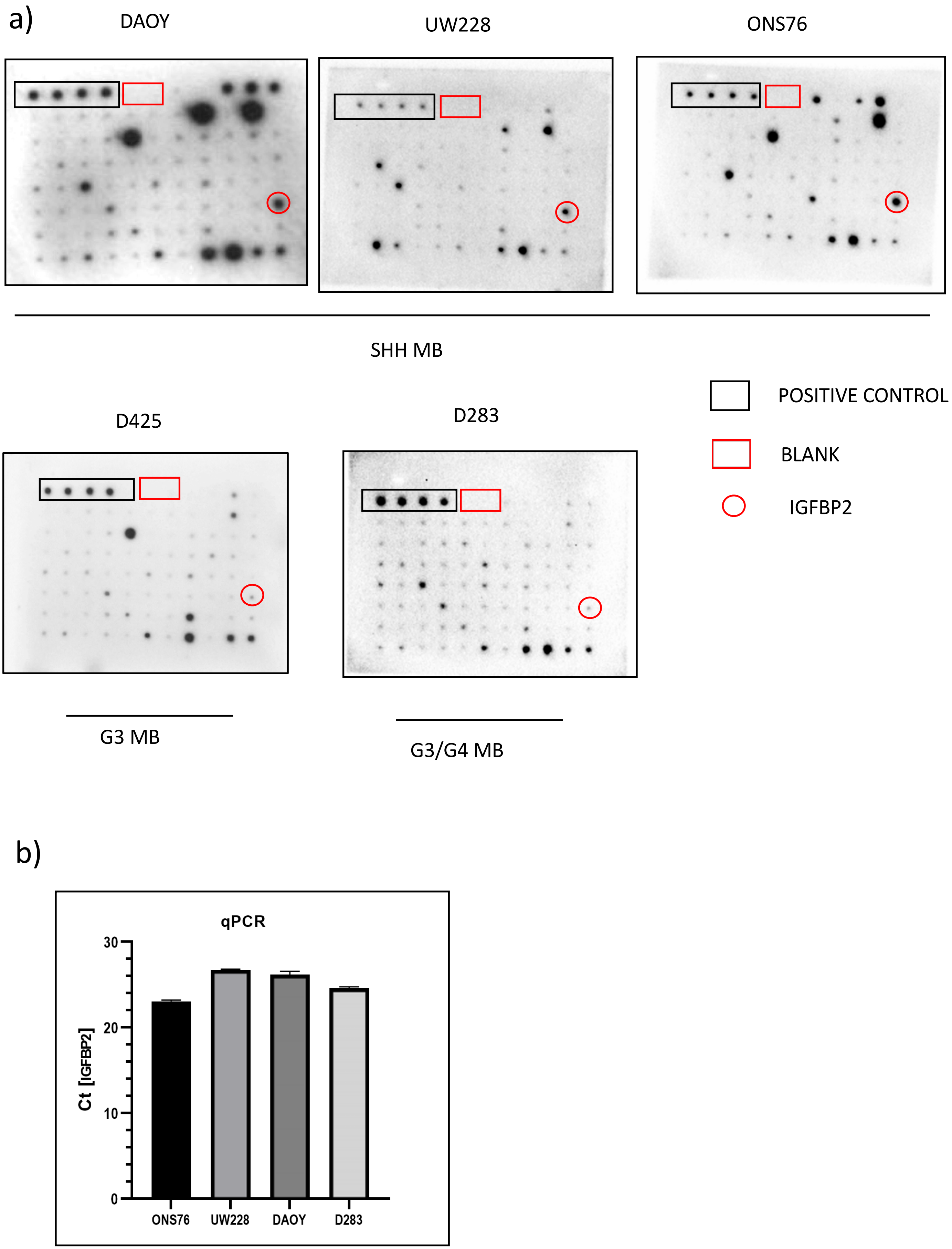

Kunhiraman Fig S2

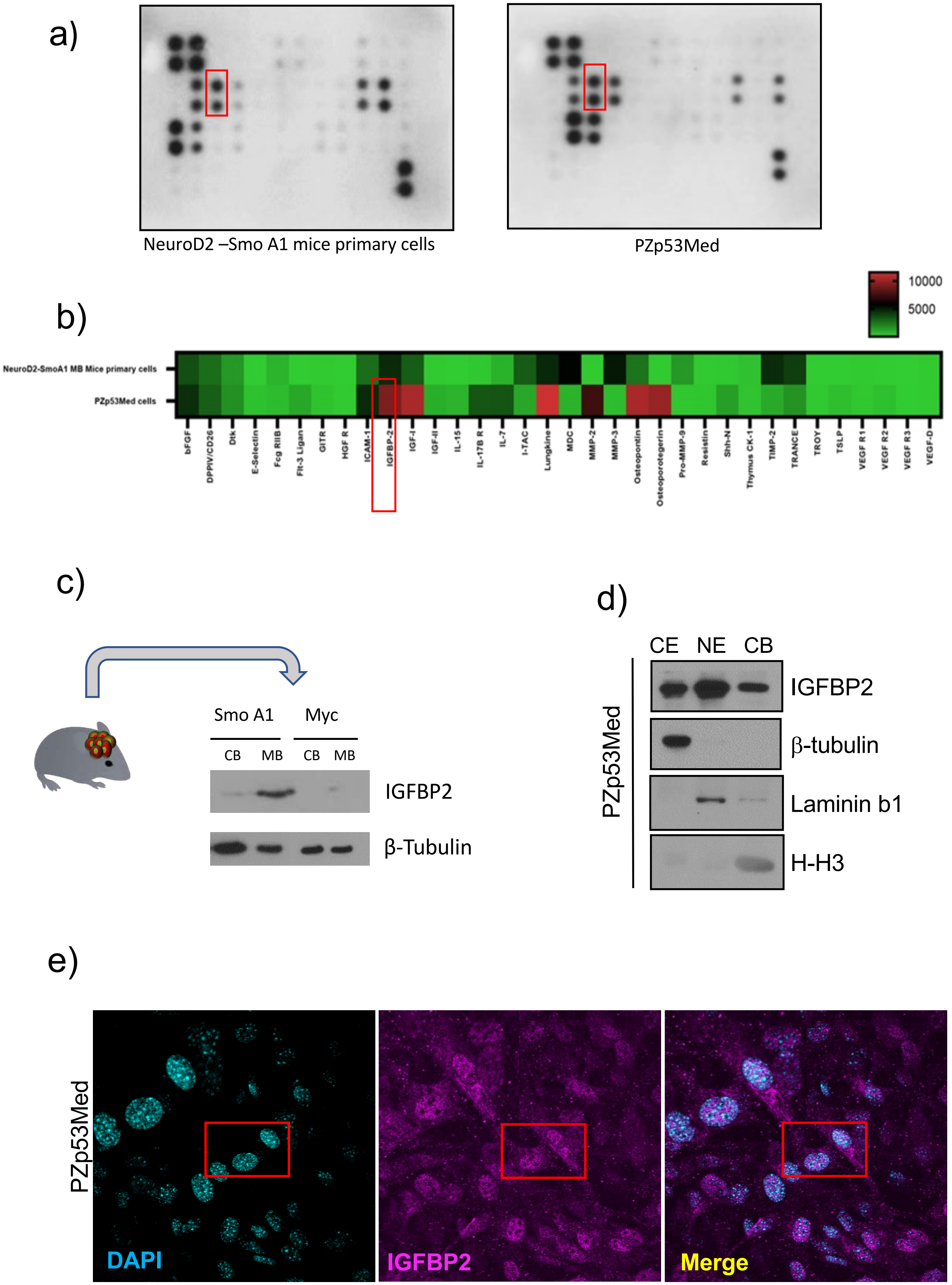

Kunhiraman Fig S3

a)

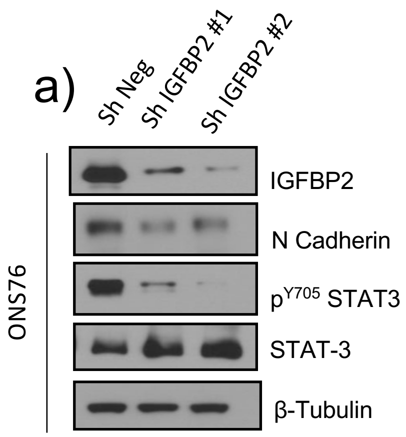

b)

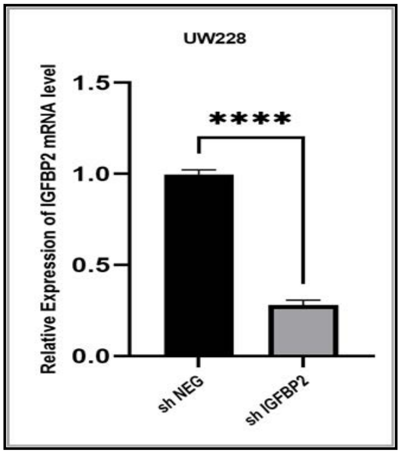

c)

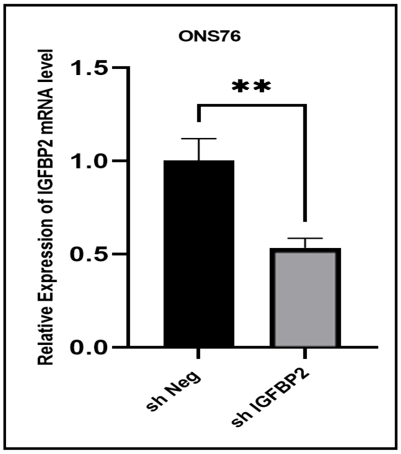

d)

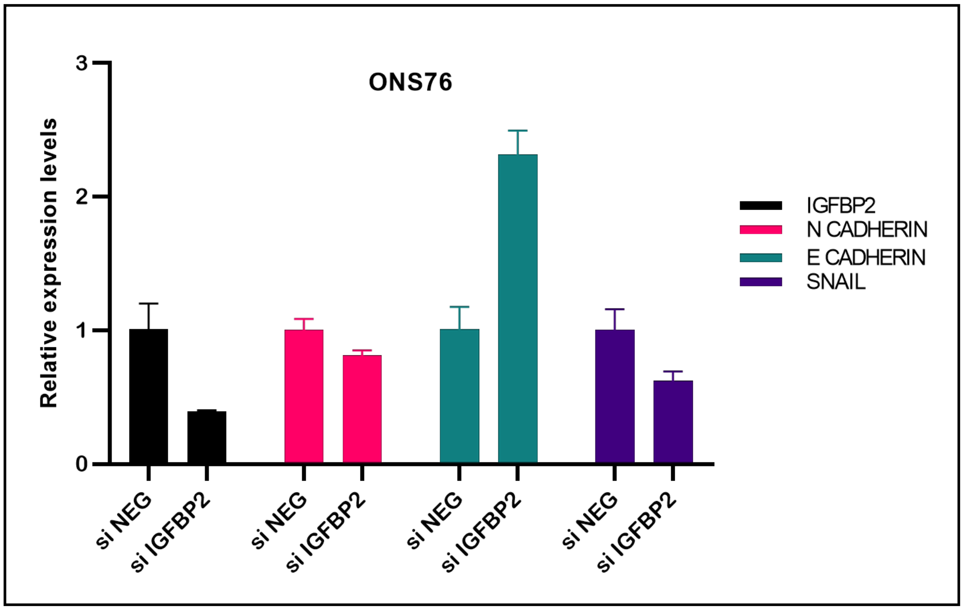

Kunhiraman Fig S4

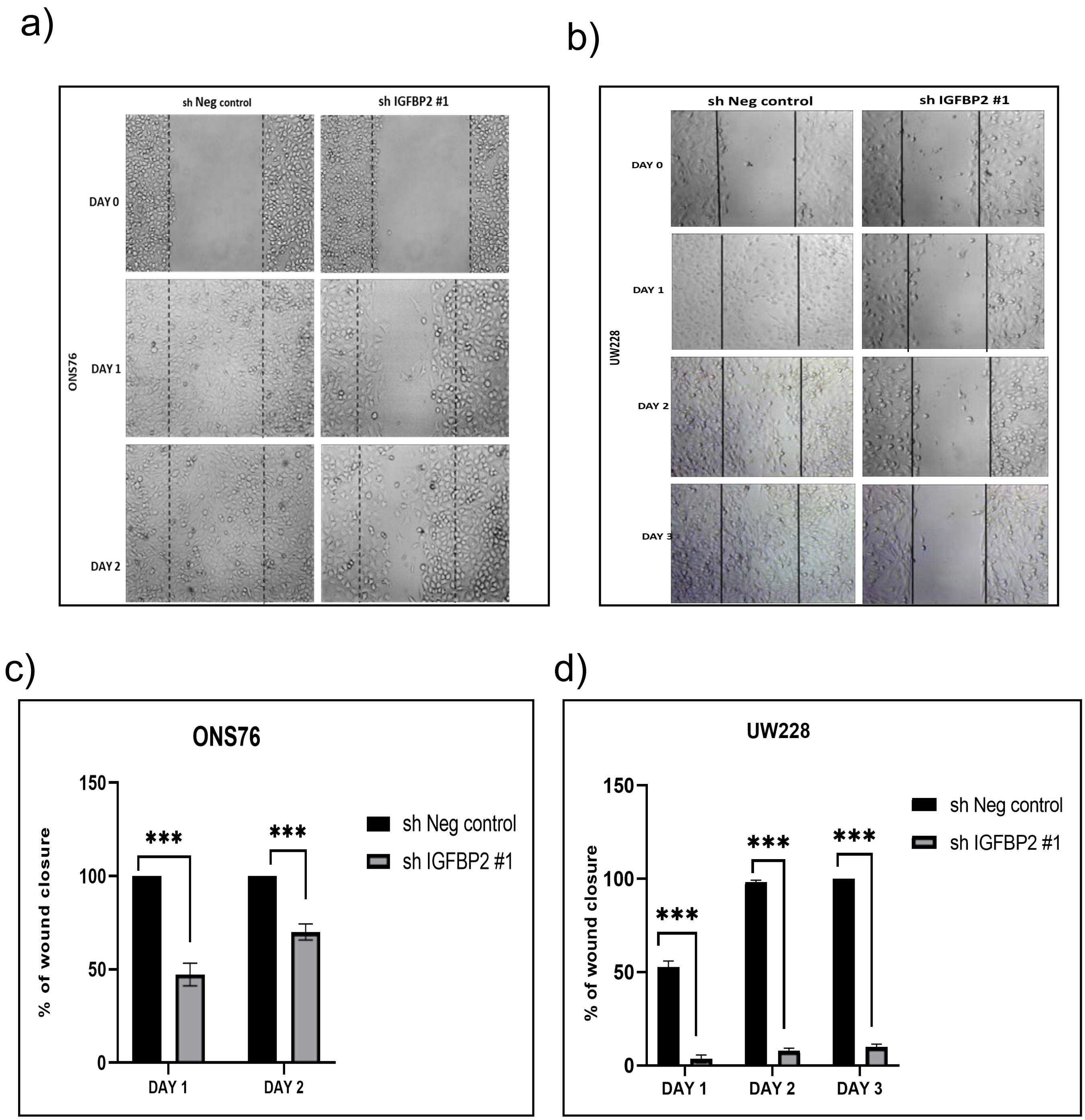

Kunhiraman Fig S5

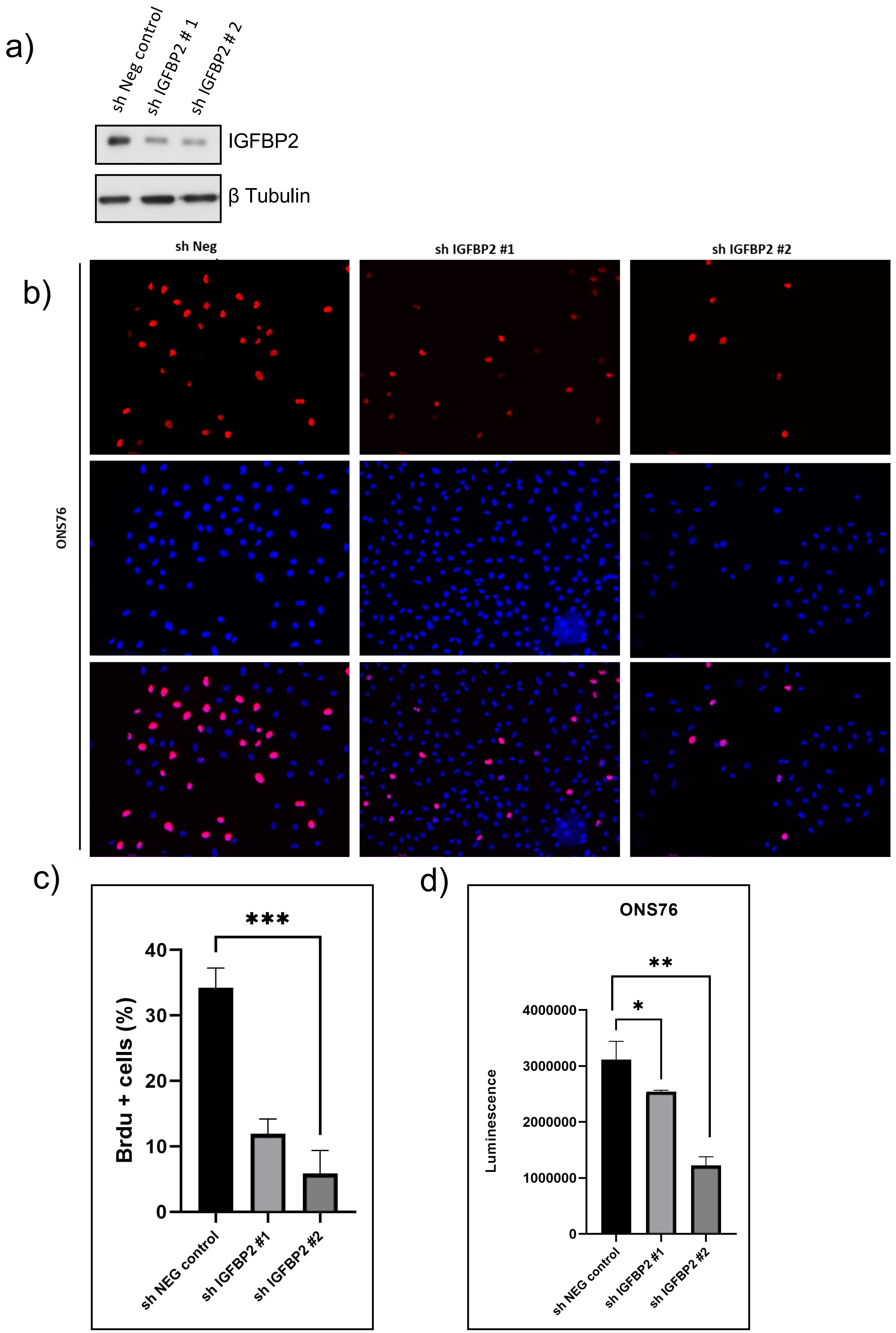

Kunhiraman Fig S6

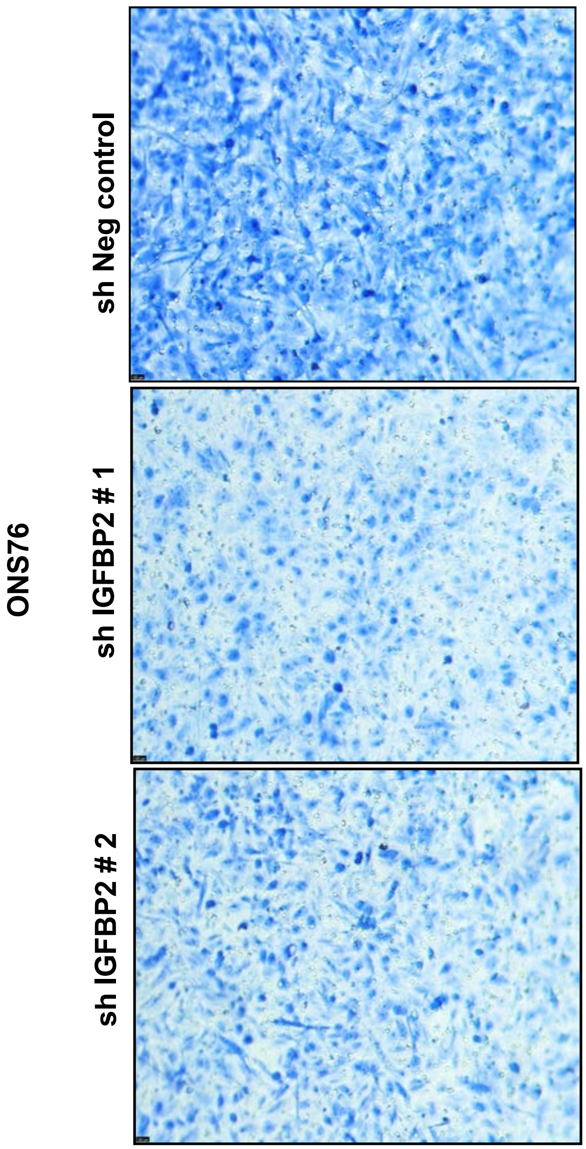

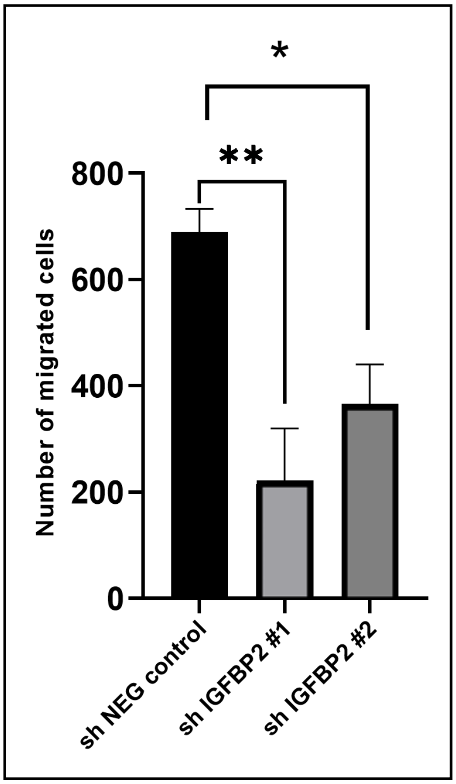

Kunhiraman Fig S7

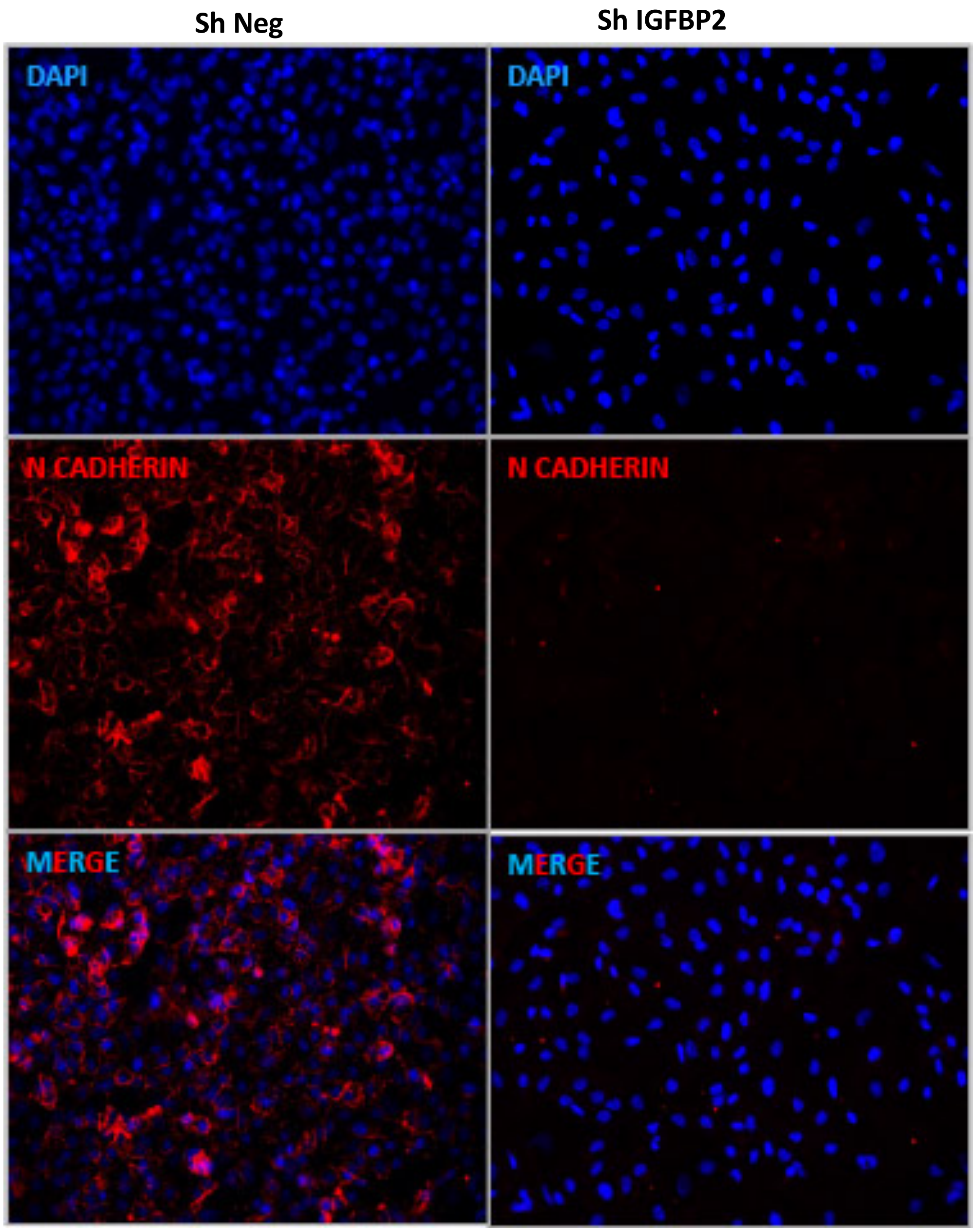

Kunhiraman Fig S8

a)

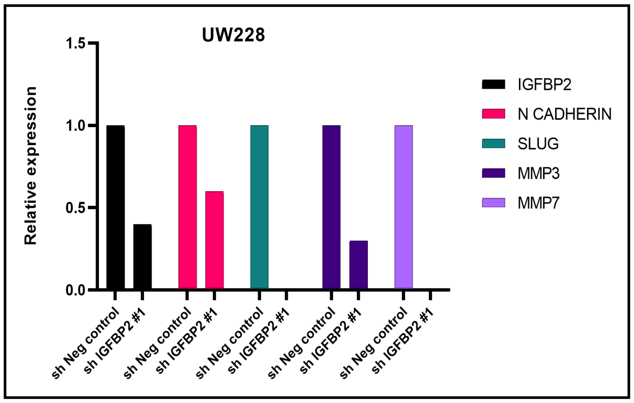

b)

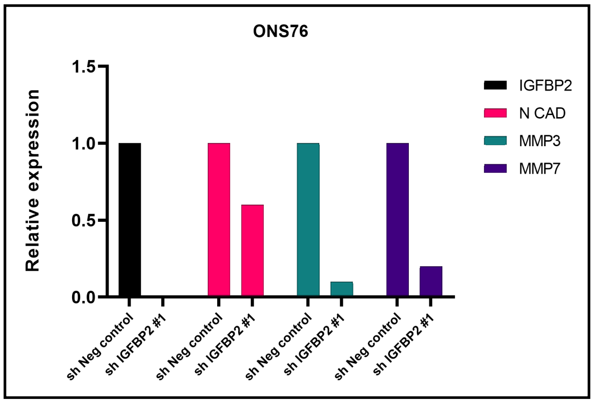

c)

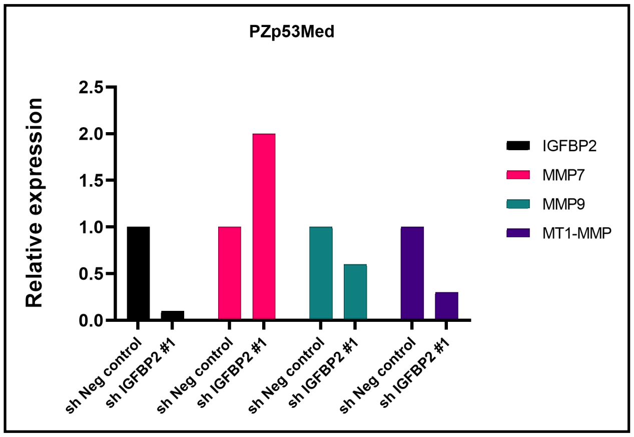

d)

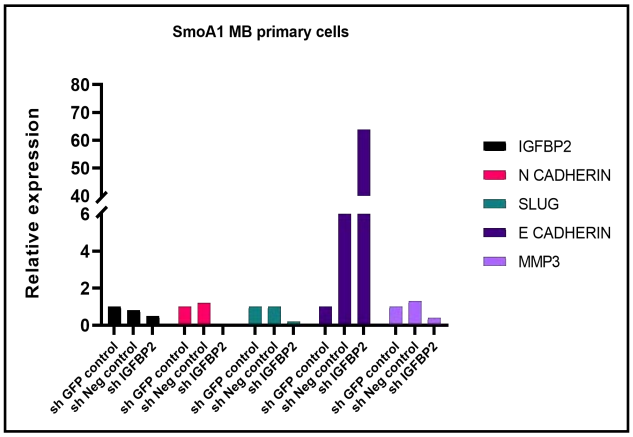

Kunhiraman Fig S9

a)

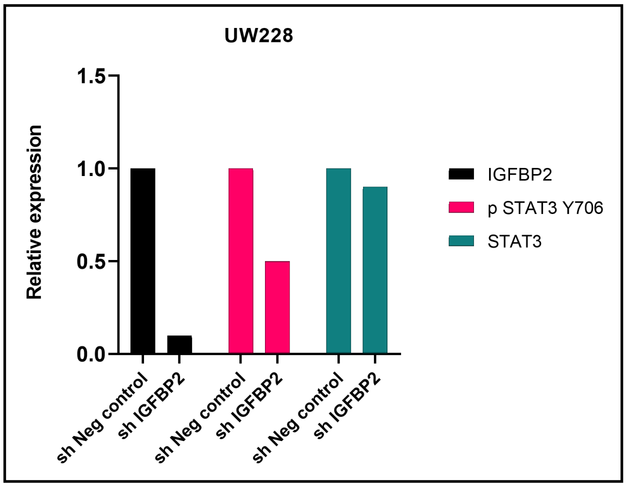

b)

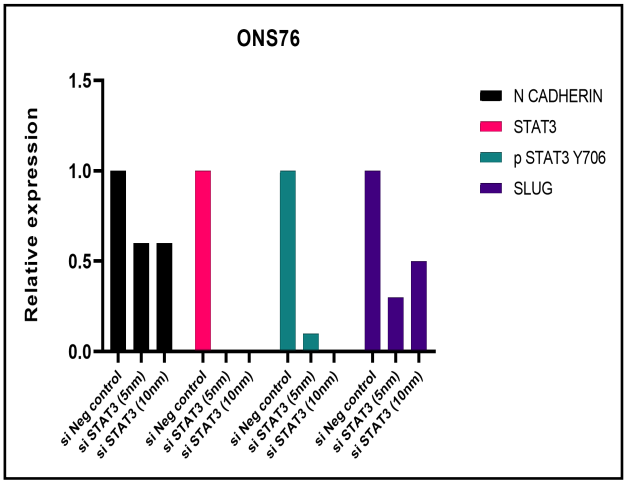

c)

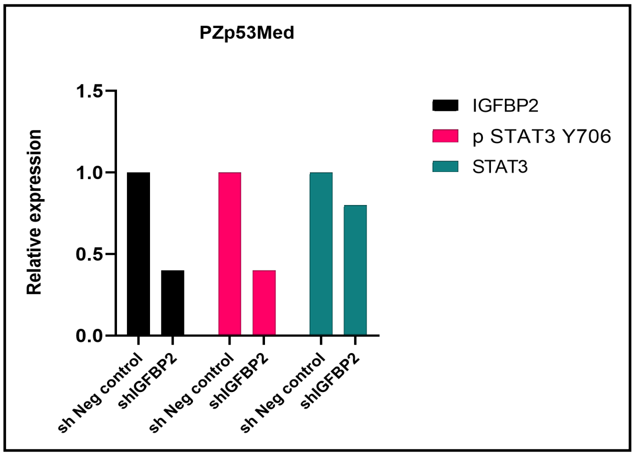

d)

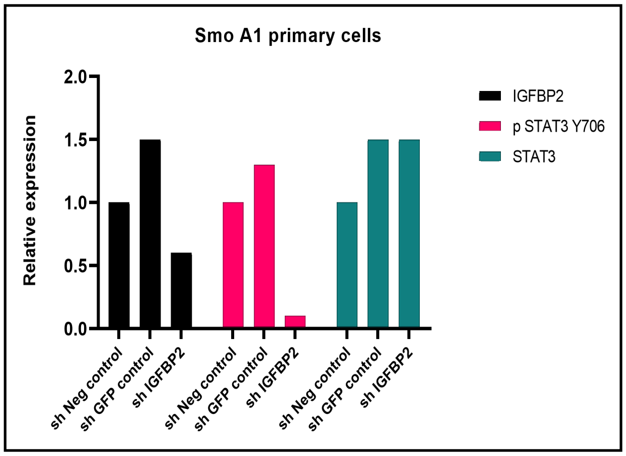

Kunhiraman Fig S10

a)

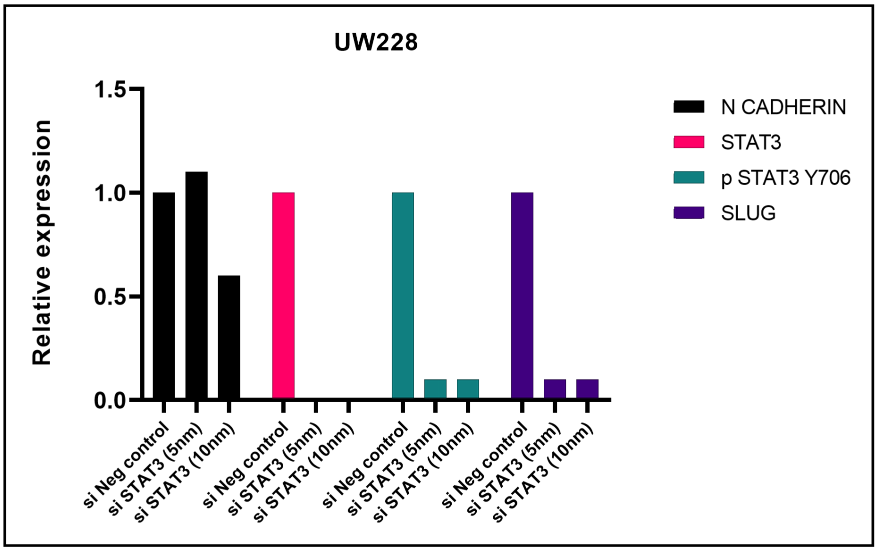

b)

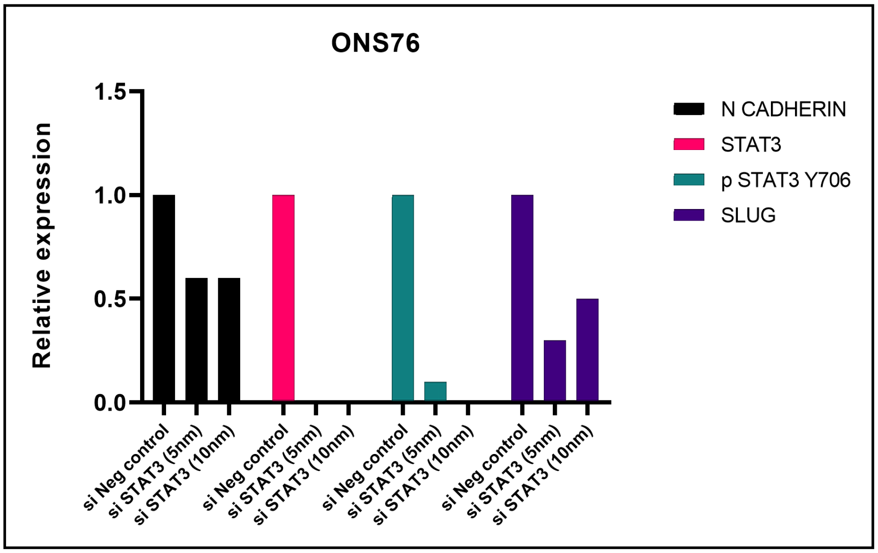

Kunhiraman Fig S11

a)

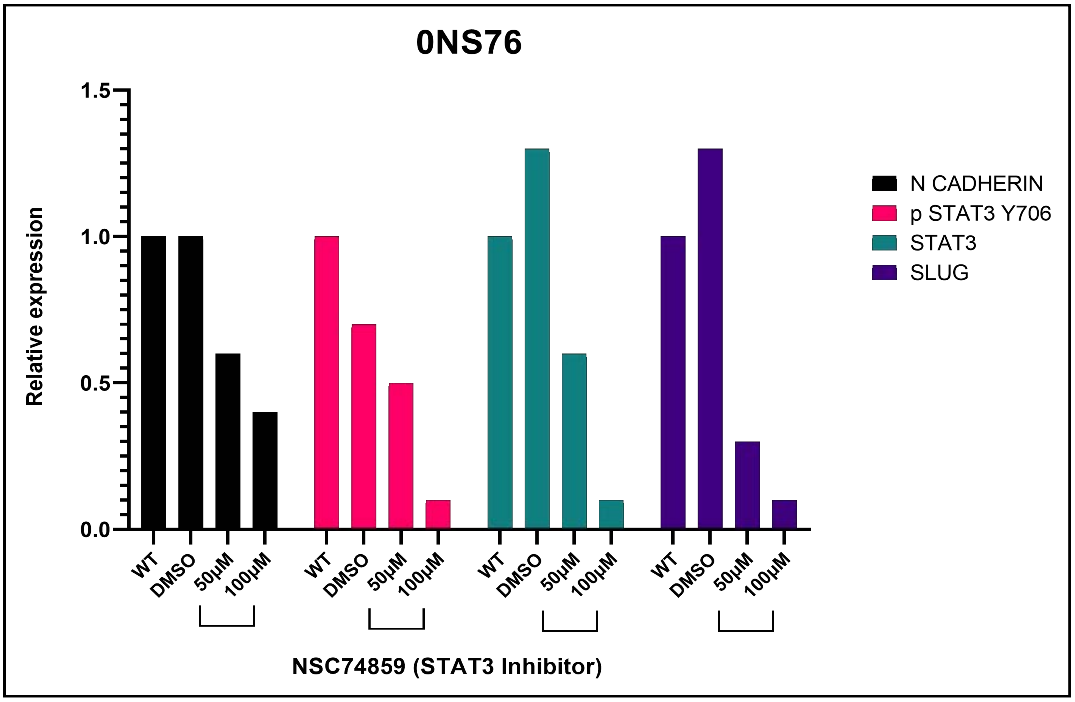

b)

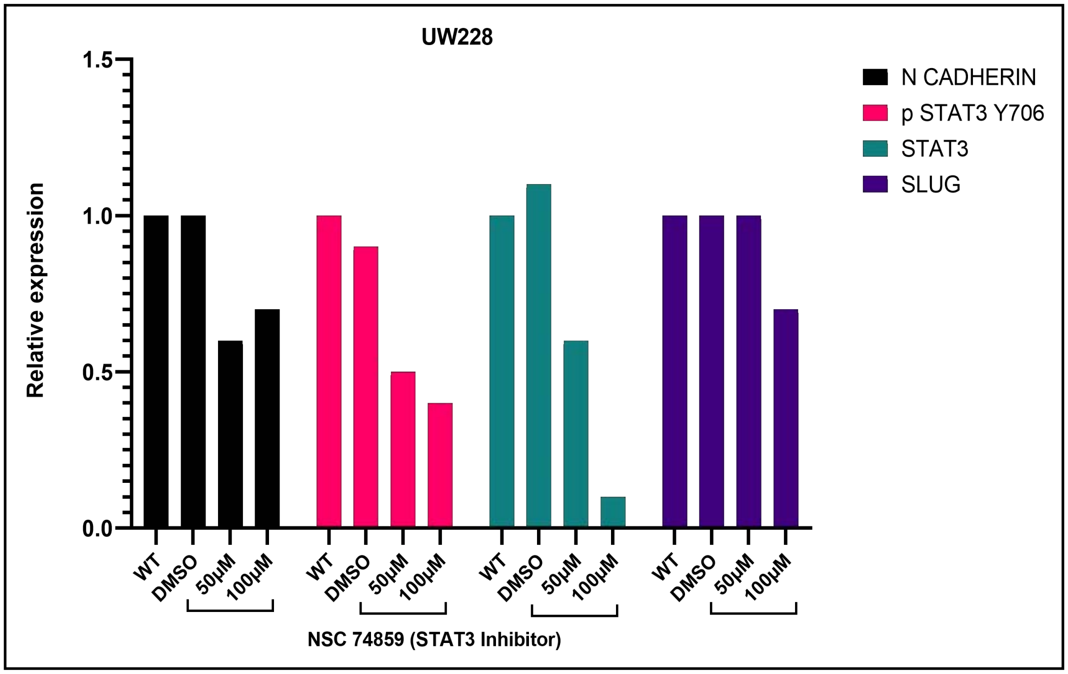

Kunhiraman Figure S12

a)

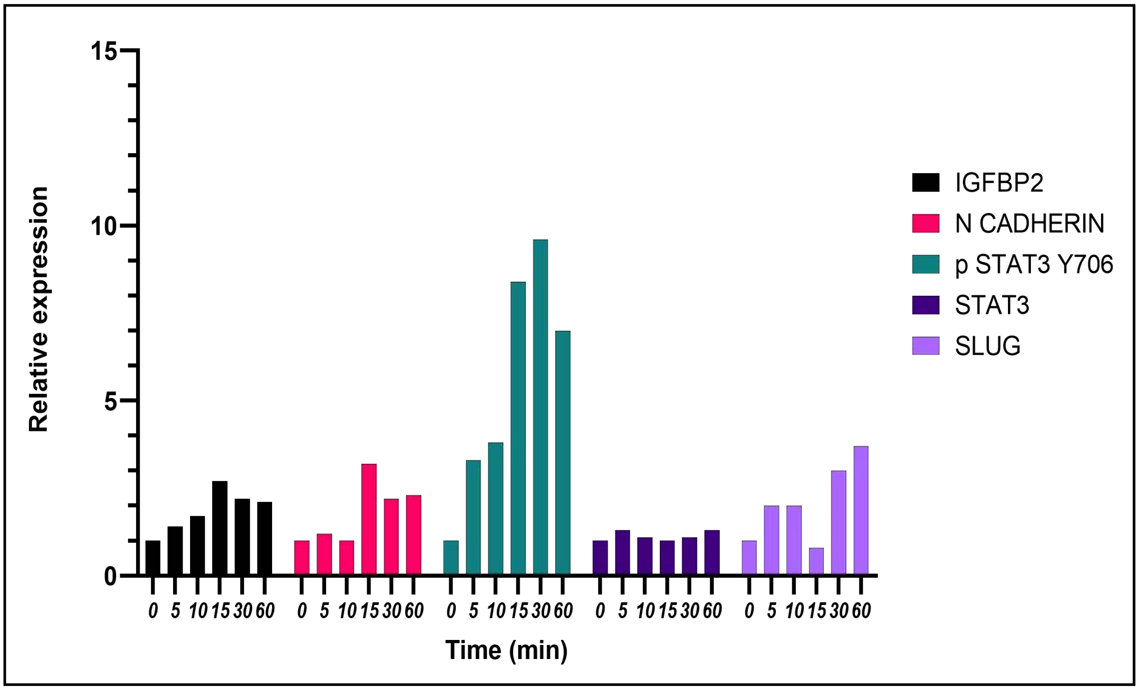

b)

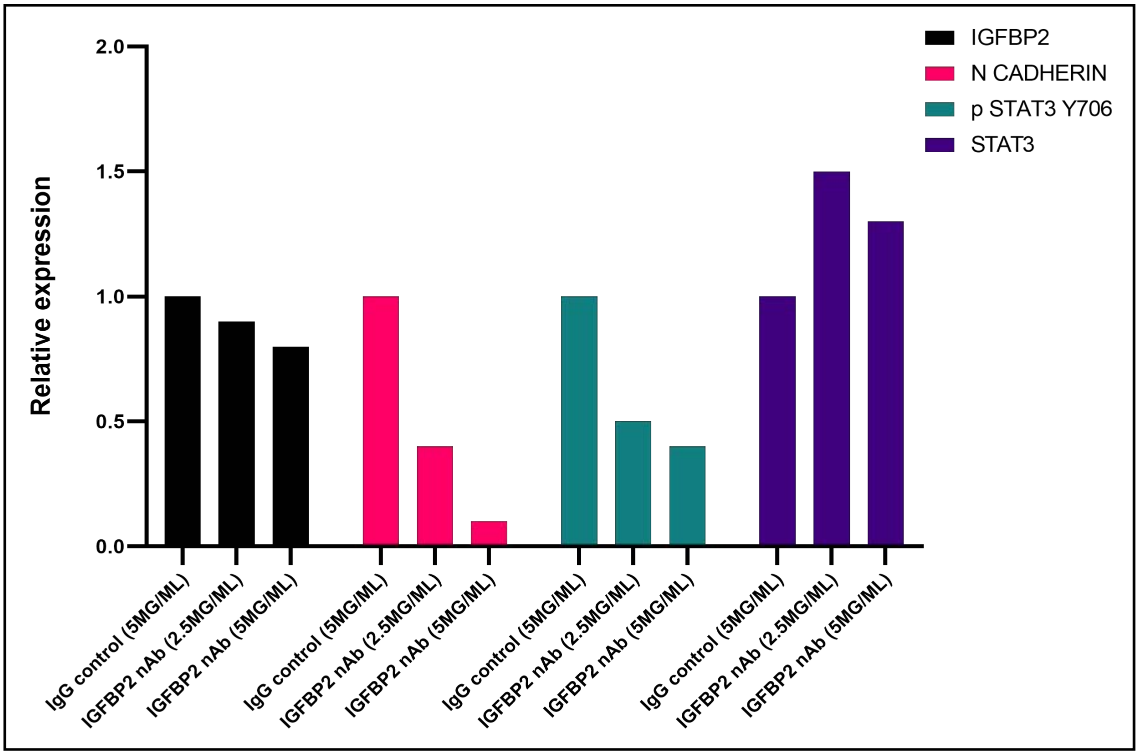

Kunhiraman Figure S13

a)

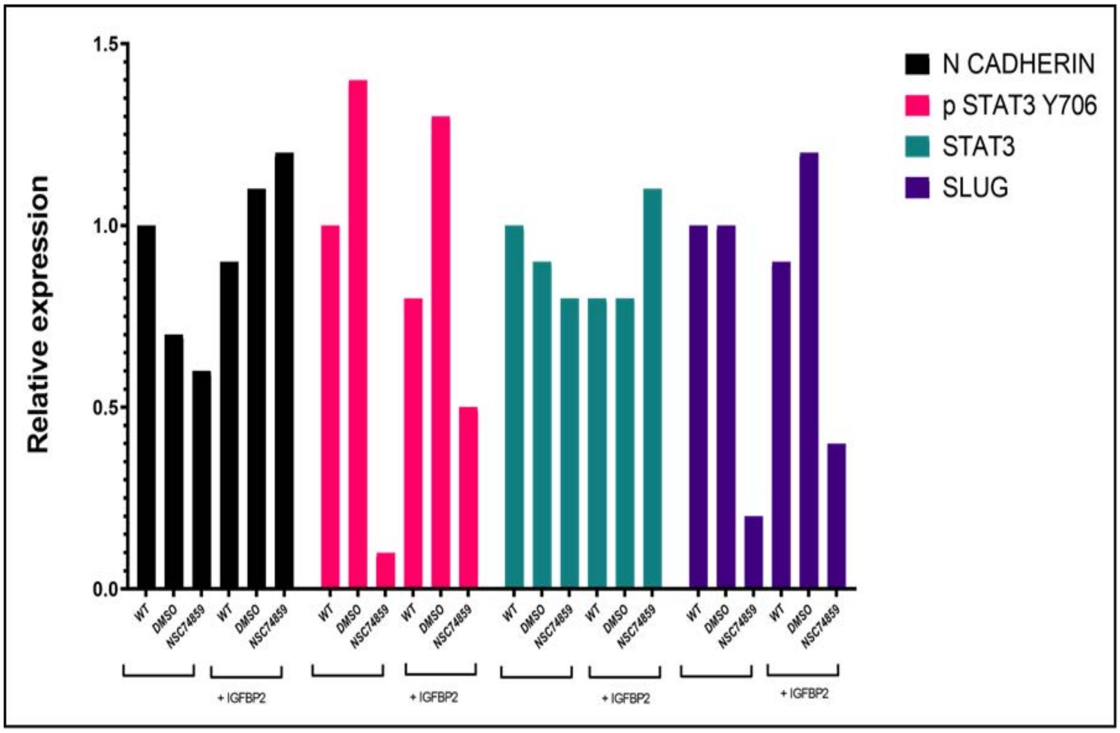

b)

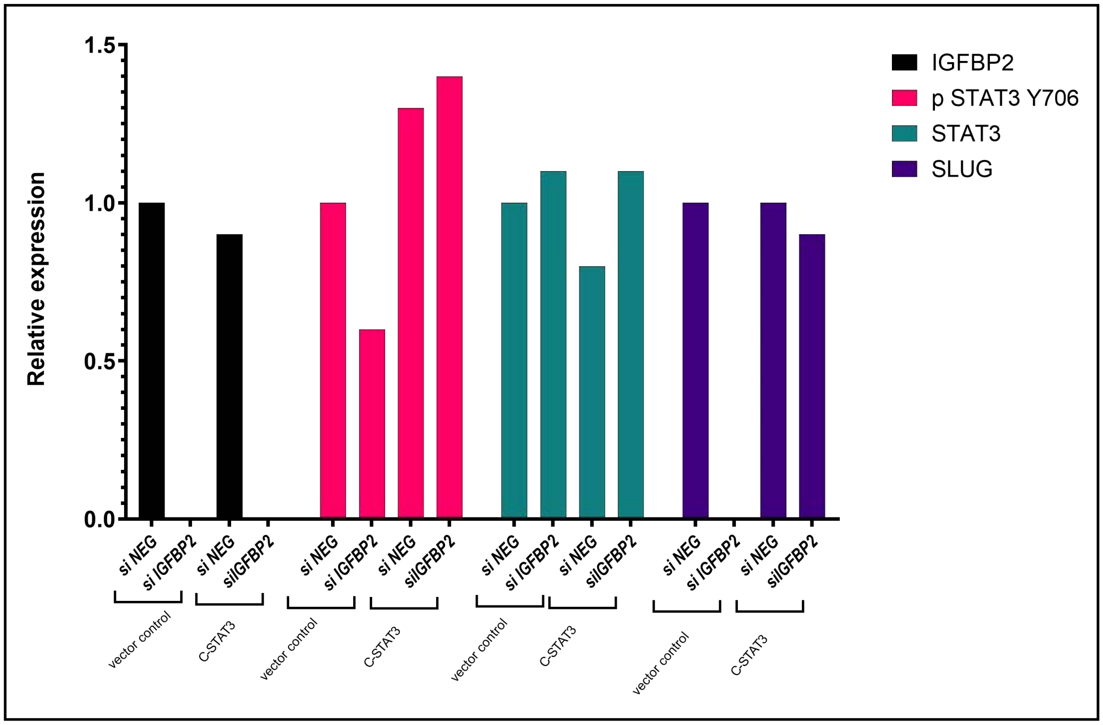

Kunhiraman Fig S14

Medulloblastoma stem cells isolated from NeuroD2: Smo/A1 mouse Medulloblastoma primary tumor.

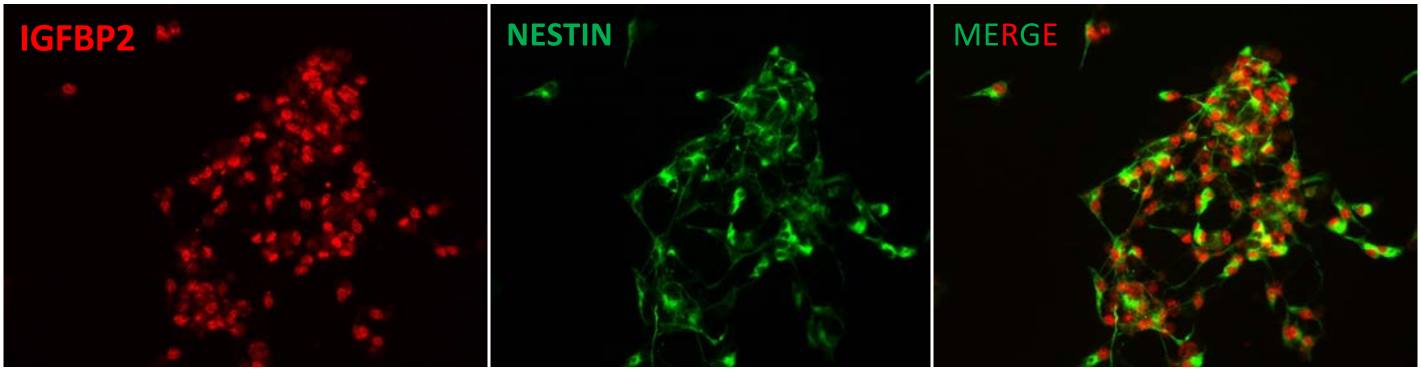

Kunhiraman Fig S15

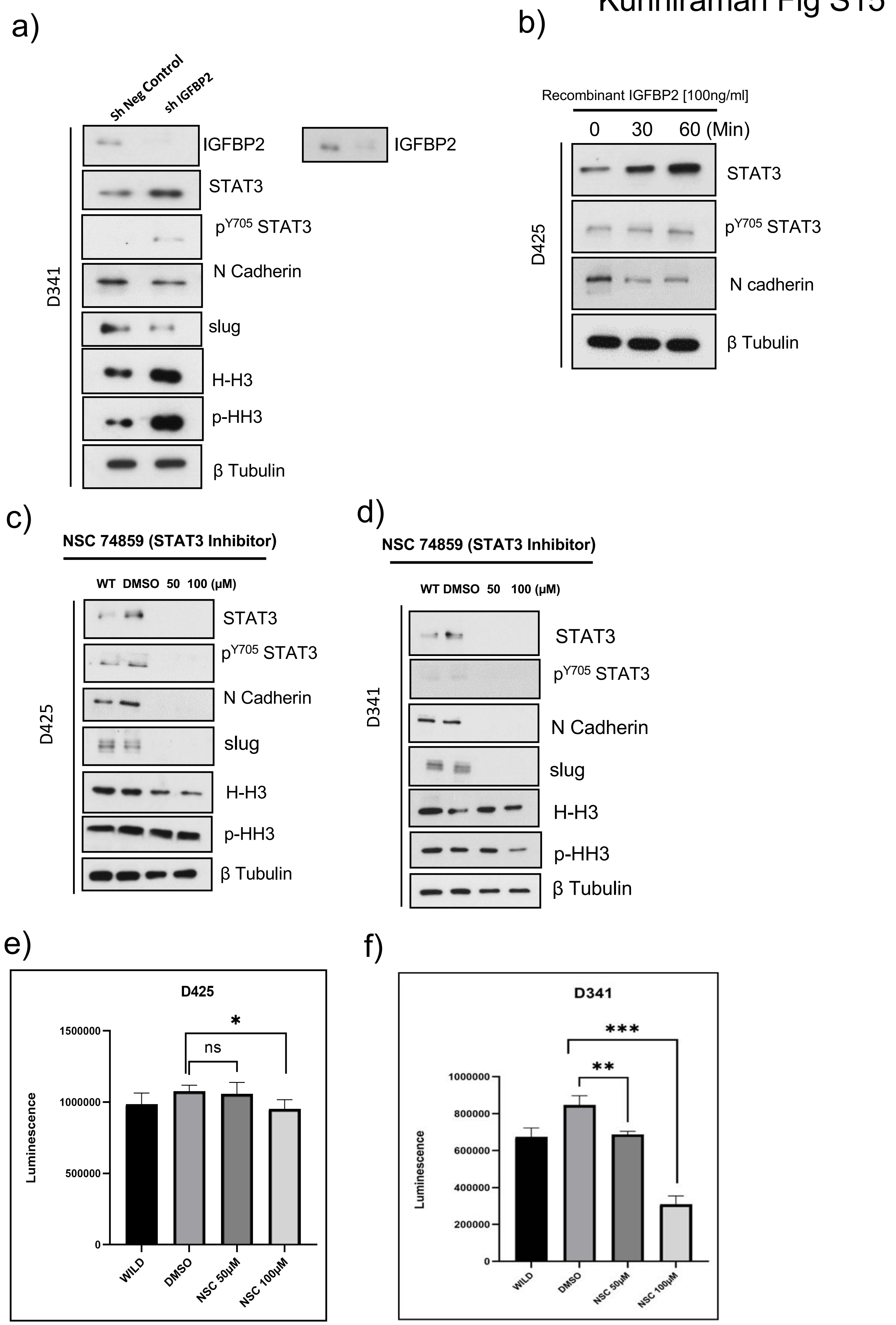

List of qPCR Primers

**Target Name**	**Forward primer 5'-3'**	**Reverse primer 5'-3'**
IGFBP2 (Mouse)	CAGACGCTACGCTGCTATCC	CCCTCAGAGTGGTCGTCATCA
Actin (Mouse)	GGCTGTATTCCCCTCCATCG	CCAGTTGGTAACAATGCCATG
GAPDH (Mouse)	AGGTCGGTGTGAACGGATTTG	TGTAGACCATGTAGTTGAGGTCA
IGFBP2 (Human)	TTGCGTCTGGCGCGT	CATCCGGAAGGCGCATGG
IGFBP2 (Human)	TGGCGATGACCACTCAGAAG	ATACCCGACTTGAGGGGCT
ACTIN (Human)	GGGCATGGGTCAGAAGGATT	TCGATGGGGTACTTCAGGGT
E cadherin (Human)	ATTTTTCCCTCGACACCCGAT	TCCCAGGCGTAGACCAAGA
N cadherin (Human)	CCATCAAGCCTGTGGGAATC	GCAGATCGGACCGGATACTG
SNAIL1(Human)	ACTGCAACAAGGAATACCTCA	GCACTGGTACTTCTTGACATCTG
